# A high-resolution dataset on the plastic material flows in Switzerland

**DOI:** 10.1016/j.dib.2022.108001

**Published:** 2022-03-02

**Authors:** Magdalena Klotz, Melanie Haupt

**Affiliations:** ETH Zurich, Institute of Environmental Engineering, John-von-Neumann Weg 9, Zurich 8093, Switzerland

**Keywords:** Plastics, Material flow analysis, Environmental assessment, Recycling, System modeling, Polymers, ABS, acrylonitrile butadiene styrene, AC, air conditioning, B&C, building and construction, C&I, commercial and industrial, CE, consumer electronics, EE, electrical and electronic, EEE, electrical and electronic equipment, ELV, end-of-life vehicle, EoL, end-of-life, HDPE, high-density polyethylene, HH, household, HIPS, high-impact polystyrene, ICT, information and communication technology, Intl., international, LDPE, low-density polyethylene, NIR, near-infrared, OEM, original equipment manufacturer, PA, polyamides, PC, polycarbonates, PET, polyethylene terephthalate, PP, polypropylene, PS, polystyrene, PTTs, pots, trays and tubs, PUR, polyurethanes, PVC, polyvinylchloride, RESH, shredder light fraction, WEEE, waste electrical and electronic equipment, WTE, waste-to-energy

## Abstract

A material flow analysis of the main plastic types used and arising as waste in Switzerland in 2017 is conducted, including consideration of stock change. Seven main plastic application segments are distinguished (packaging; building and construction; automotive; electrical and electronic equipment; agriculture; household items, furniture, leisure and others; and textiles), further divided into 54 product subsegments. For each segment, the most commonly used plastic types are considered, in total including eleven plastic types (HDPE, LDPE, PP, PET, PS, PVC, ABS, HIPS, PA, PC, and PUR). All product life cycle stages are regarded, including the determination of the product subsegments in which the individual post-consumer secondary materials obtained from mechanical recycling are applied. The underlying data are gathered from official statistics and administrative databases, scientific literature, reports by industry organizations and research institutions, websites, and personal communication with stakeholders. The compiled data are then reconciled. All flow data are provided and depicted in two Sankey diagrams: one diagram shows the material flows on a product-subsegment level and the second one on a plastic-type level. Users may retrieve the data with a script and transfer them into a relational database. The present material flow analysis data are used as a basis for the scenario analysis in Klotz et al. [1]. Besides scenario modelling, the data can be used in conducting life cycle assessments. Both utilizations can serve as a support for designing future plastic flow systems.

**Specifications Table**

**Table:** 

Subject	Environmental engineering
Specific subject area	Application of material flow analysis on national plastic flows with a system perspective (from production to waste management)
Type of data	Tables Figures, including Sankey diagrams Charts
How data were acquired	Raw data gathered from expert interviews, databases, literature review, reports and websites processed within a material flow analysis
Data format	Raw Reconciled Analyzed
Description of data collection	A material flow analysis was conducted for plastics consumed and arising as waste in Switzerland in 2017. The underlying data were gathered from personal communication with stakeholders, databases, scientific literature, reports and websites. The compiled data were reconciled, and missing flows were calculated using a mass balance approach.
Data source location	Institution: ETH Zurich Country: Switzerland Primary data sources (selection): [2], [3], [4], [5], [6], [7], [8], [9], [10], [11], [12]
Data accessibility	With the article
Related research article	M. Klotz, M. Haupt, S. Hellweg, Limited Utilization Options for Secondary Plastics May Restrict Their Circularity, Waste Manag., 141 (2022), 251*–*270. https://doi.org/10.1016/j.wasman.2022.01.002[1]

## Value of the Data

• The provided data can be used in designing a plastic waste management system for Switzerland, for conducting a related life cycle assessment, or as a methodological reference for similar studies.
• Stakeholders who can directly benefit from the provided data include policy-makers, as well as institutions and consultancies aiming to develop plastic waste management systems. The data can also be of use to scientists who are interested in performing similar analyses or life cycle assessments.
• For gaining further insights, for example regarding the environmental impacts of different parts of the system or of different waste management options, the provided data can be used as a basis for modelling future scenarios and conducting prospective life cycle assessments.

## 1 Data Description

The presented data comprises three parts. The content and structure of these are described in this chapter. Additionally, a way for transferring the material flow data into a relational database is described.

### 1.1 Database

The database (referred to as such in the following) contains all material flow data, with related calculations and data sources. It is stored as an Excel workbook consisting of various sheets.

**Fig. 1:**
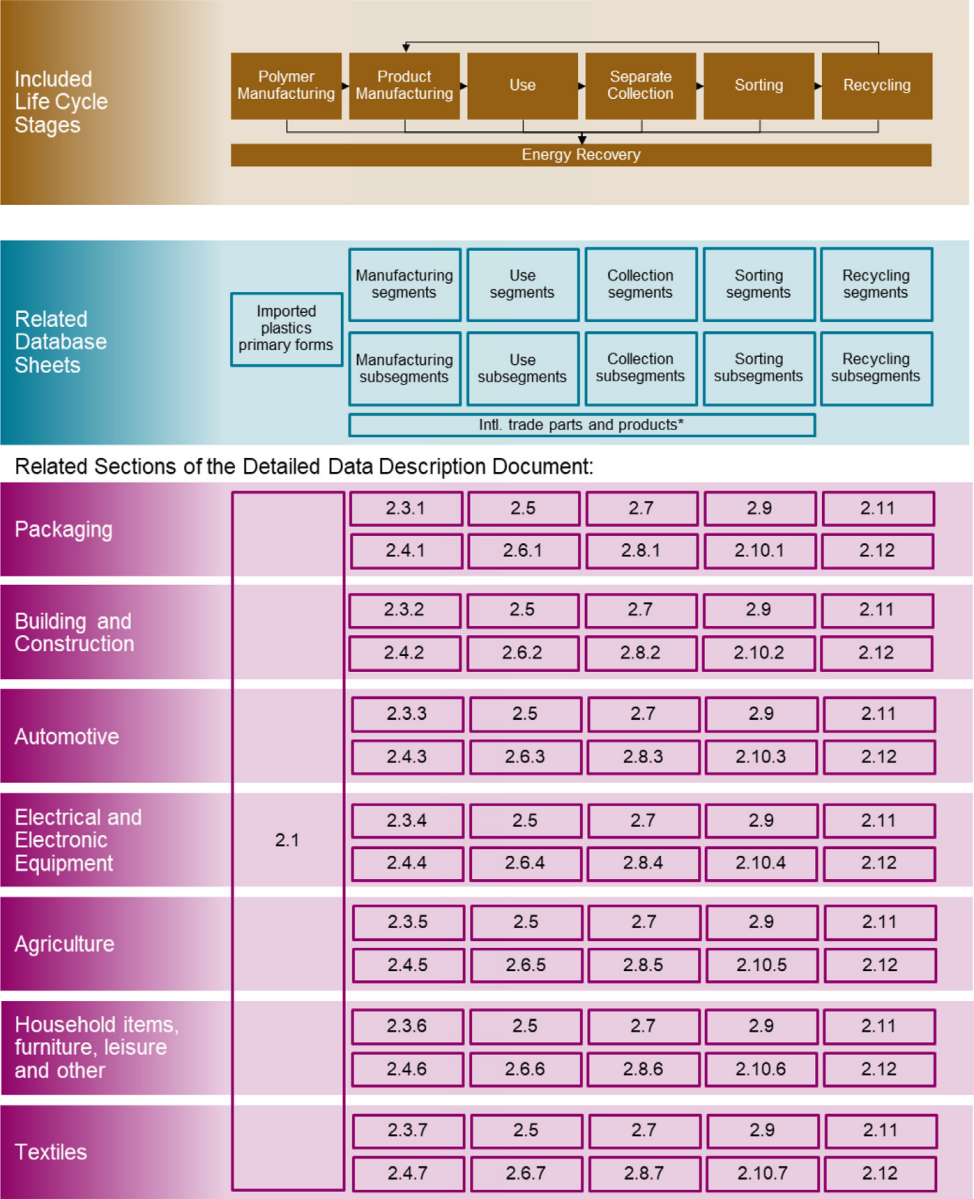
Overview of included life cycle stages, as well as corresponding database sheets and sections of the detailed data description document. Use and separate collection refer to the respective processes taking place in Switzerland, while the considered polymer and product manufacturing, sorting, recycling and energy recovery processes at least partly take place abroad. Chemical recycling processes, which allow to gain a product that can be used for polymer manufacturing, were not relevant for the research work; neither was landfilling, as combustible waste must be incinerated in Switzerland if no material recycling takes place [13].

The flow data are stored in sheets of the database according to the plastic life cycle (Figure 1). Besides the sheets relating to the material flows in the different life cycle stages, there are further sheets in the database. The General information sheet gives relevant information on how to read the data in the database. It includes all abbreviations, an explanation of the color coding, a glossary, general explanations to certain product segments and life cycle stages, important information regarding the connection of the database with the Sankey diagrams, and a possible way to transfer of the data into a relational database [1]. The References sheet contains all literature references mentioned in the database, and personal data of the people who provided information via personal communication. The variable parameters sheet contains parameters that were used in the flow calculations, together with related sources and critical discussion. Several help sheets of grey tab color document auxiliary calculations for the material flows. For the connection with the Sankey diagrams, all relevant flow data are gathered on additional sheets (see Section 1.3). Additionally, all flow data are compiled in a structured form on specific sheets from which the data can be read out and written into a relational database, as done in [1] (see Section 1.4).

### 1.2 Detailed data description document

In the detailed data description document (which it is referred to as in the following and in the database), the data sources and calculations for data compilation and reconciliation are described and discussed. Information on the collection, sorting and recycling processes in place is provided.

The description is structured according to the product life cycle (Figure 1). Each of the Sections 2.1 to 2.12 of the detailed data description document refers to one sheet of the database.

### 1.3 Sankey diagrams

Two Sankey diagrams depicting the material flow data are provided:

• Sankey diagram on the plastic-type level: This flow diagram depicts the material flow data from the database on the level of plastic types. The distinguished plastic types are provided in Section 2.2. The diagram is provided in PDF and .sankey format.
• Sankey diagram on the product-subsegment level: This flow diagram depicts the material flow data from the database on a product subsegment level. A list of the distinguished product segments and subsegments is provided in Section 2.2. The diagram is provided in PDF and .sankey format.

For depicting the material flow data in the database as Sankey diagrams, specific database sheets were created, to which the Sankey diagrams are linked. The respective sheets are located after the sheets regarding the life cycle stages and are named according to the product segments. The sheets linked to the Sankey diagram on plastic-type level contain the term “polymers_diagram”, and the sheets linked to the Sankey diagram on subsegment level contain the term “product_diagram”.^1^ The flows into polymer manufacturing and from polymer manufacturing to product manufacturing were calculated via the mass balances of the respective processes, assuming that no stock changes occur. For the manufacturing taking place abroad of products imported into Switzerland, the manufacturing losses were not modelled, i.e. the amount of input materials into manufacturing corresponds to the amount of manufactured products. In reality, an additional input of virgin materials corresponding to the manufacturing losses into the respective process is needed. For calculating the flows from polymer manufacturing to product manufacturing, relating to virgin plastics, the amounts of secondary plastics used in product manufacturing according to the present model were considered. Specifically, the determined secondary material flows were subtracted from the total demand in product manufacturing to get the virgin material input flows, i.e., a 1:1 substitution was assumed. This does not necessarily reflect the reality in all cases, but was used for a first best-case assessment.

### 1.4 Modality for a transfer of the material flow data into a relational database

The presented material flow data, for a scenario analysis [1], was transferred from the Excel database into a relational database in MySQL, which was then accessed to retrieve data and conduct calculations, with Python. The MFA data can also be retrieved from the Excel database by other researchers in a similar way. For this purpose, specific sheets were added in the Excel workbook, each containing structured data that correspond to tables of a relational database. The names of these sheets start with “db_”. The relations among the individual tables are provided using unique identifiers. Foreign keys can be applied to ensure consistency. The structure of a relational database seems suitable for MFAs; the Open Dynamic Material Systems Model (ODYM) framework developed by Pauliuk and Heeren [14] was a move in a similar direction.

## 2 Experimental Design, Materials and Methods

### 2.1 Methods

A static material flow analysis (MFA) considering stock change was conducted. The system boundaries are described in Section 2 of this chapter.

The underlying data for the MFA were gathered from databases from relevant organizations, official statistics offices, studies conducted by institutes and consultancies, scientific literature, reports from industry organizations, information from companies’ websites, and personal communication with stakeholders. This research work builds on the two most recent studies of the plastic material flows in Switzerland, i.e. Kawecki et al [2]. and Schelker and Geisselhardt [3]. The material flow data were reconciled and compared to relevant studies wherever possible. Details on the data collection and reconciliation process are provided in the detailed data description document.

The data compilation was done in Excel. The material flows are depicted in Sankey diagrams, which were created with e!Sankey 4 pro and are directly linked to the Excel workbook. The flow data was prepared to be transferred from Excel to a relational database.

### 2.2 System definition

The geographical boundary of the material flow analysis is constituted by plastics used and arising as waste in Switzerland. In order to include the complete material life cycle, relevant disposal processes taking place abroad were included. The temporal boundary was the year 2017.

The plastic types considered in the material flow analysis are listed in Table 1 and on the database sheet labeled Plastic types. For each product segment (Table 2), individual plastic types were included to cover the largest share of all plastics used, whereby some plastic types were considered for all segments. The estimated compositions of the individual product segments and the approximately covered share of each product segment by the considered plastic types are provided on the same sheet Plastic types. The shares of different plastic types used in different product segments were also used to estimate the amounts of different plastic types in certain products where more specific information was not available.

**Table 1:** Plastic types considered in the material flow analysis

	Plastic type denomination	Abbreviation
Commodity plastics	High-density polyethylene	HDPE
	Low-density polyethylene	LDPE
	Polyethylene terephthalate	PET
	Polypropylene	PP
	Polystyrene	PS
	Polyvinylchloride	PVC

Technical plastics	Acrylonitrile butadiene styrene	ABS
	High-impact polystyrene	HIPS
	Polyamides	PA
	Polycarbonates	PC
	Polyurethanes	PUR

**Table 2:** Products segments and subsegments considered in the material flow analysis (PTTs: pots, trays and tubs; C&I: commercial and industrial; B&C: building and construction; HH: household; AC: air conditioning; ICT: information and communication technology; CE: consumer electronics; EEE: electrical and electronic equipment)

Product segment	Product subsegment
Packaging	Food films
	Food bags
	Food bottles
	Food PTTs
	Food other
	Consumer non-food films
	Consumer non-food bags
	Consumer non-food bottles
	Consumer non-food PTTs
	Consumer non-food other
	Non-consumer packaging C&I - manufacturing - films
	Non-consumer packaging C&I - manufacturing - rigids
	Non-consumer packaging C&I - retail - films
	Non-consumer packaging C&I - retail - other
	Non-consumer packaging C&I - hospitality - films
	Non-consumer packaging C&I - hospitality - bottles
	Non-consumer packaging C&I - hospitality - PTTs
	Non-consumer packaging C&I - hospitality - other
	Non-consumer packaging B&C - films
	Non-consumer packaging B&C - rigids
	Non-consumer packaging agriculture - films
	Non-consumer packaging agriculture - rigids

Building and Construction	Pipes and ducts
	Thermal insulation
	Flooring
	Window profiles
	Roof lining
	Other B&C products

Automotive	

Electrical and Electronic Equipment	Large HH appliances
	Cooling, refrigerating and AC devices
	Small HH appliances
	ICT equipment and CE
	Other EEE

Agriculture	Agricultural films - silage
	Agricultural films - greenhouse
	Agricultural films - mulch
	Agricultural films - other
	Agricultural pipes
	Other agricultural products
Household items, furniture, leisure and others	Household items
	Toys
	Furniture
	Sports items
	Medical and hygiene items
	Other products

Textiles	Apparel
	Household textiles
	Technical textiles - textile flooring
	Technical textiles - textile furniture
	Technical textiles - mobility textiles
	Technical textiles - agrotextiles - agricultural nets
	Technical textiles - agrotextiles - other agrotextiles
	Technical textiles - other technical textiles

Plastics can generally be categorized based on different characteristics such as functional groups present in the polymer^2^ or monomer^3^, temperature behavior^4^, use^5^, material origin^6^ or degradability^7^. For this research work, typical categories for plastic types were used (see [2,15]): some refer to plastics made from specific monomers (LDPE, HDPE, PET, PP, PS, PVC, ABS, HIPS), whereas the other types consist of plastics made from similar, but not identical monomers (PC, PA, PUR). The exact polymers included in each of these plastic types are specified in detail on the database sheet Plastic types. All distinguished plastic types include diverse plastics, which have varying chemical structures (chain length distribution, side chains configuration) and contain a variety of additives (see [16]). All considered plastic types are thermoplastics, except for polyurethanes (PUR), which are often thermosets. Today, more than 94% of all plastics that are produced worldwide are made from fossil feedstocks [17].

The material flows of the mentioned plastic types were further subdivided based on product groups in which they are used. To determine the scope of differentiated product segments and subsegments, different product categorization schemes applied in the fields of official statistics, trade of goods, customs, and product management on the international level were reviewed regarding their suitability [18], [19], [20], [21], [22], [23], [24], [25]. Many of the differentiated product groups of the considered schemes, however, do not contain plastic products or contain products made of other materials additionally to plastic products. It was, therefore, considered useful to rather choose product categories that are in line with commonly applied categories in plastic studies and statistics. The considered main product segments and product subsegments (see Table 2), as well as included and not included products for each subsegment are provided on the database sheet Product (sub)segments. All flows were determined on a subsegment level, except for the flows of imported electrical and electronic equipment (EEE) parts, re-used parts from waste electrical and electronic equipment (WEEE), and secondary materials from WEEE recycling, where a determination on the subsegment level was not possible due to missing data.

All main stages of the plastic life cycle, as shown in Figure 1, are included in the material flow model. For depicting the closed life cycle, the material flows of the secondary material obtained from recycling back into product manufacturing were determined.

Recycling refers to mechanical recycling. Chemical recycling processes were not applied to a relevant extent for the investigated waste flows. The definition of mechanical recycling used in this research work, as well as details on the differentiation between the sorting and recycling stages, are provided on the database sheet General information. The research work focuses on the treatment of post-consumer waste^8^, as production (pre-consumer) waste is only responsible for about 15% of the total plastics waste [26]^9^. Production losses arising in Switzerland were considered as additional input into the product manufacturing processes, i.e. the amounts of input materials are higher than the amounts of final products produced. Losses arising during installation of imported semi-finished products (e.g. cut-offs during pipe installations) were considered by directly deducing the respective share from the import amounts.

## CRediT Author Statement

*Melanie Haupt*: Conceptualization, Methodology, Supervision, Writing – review & editing; *Magdalena Klotz*: Methodology, Investigation, Data curation, Visualization, Writing – original draft preparation.

## Declaration of Competing Interest

The authors declare that they have no known competing financial interests or personal relationships which have or could be perceived to have influenced the work reported in this article.

## Appendix Supplementary materials

Database

Detailed Data Description Document

Sankey_plastics

Sankey_plastics

Sankey_products

Sankey_products

## Footnotes

1 Regarding the Sankey diagram on plastic-type level, there is one additional sheet containing “Intercon” instead of the name of a product segment in its denomination. The reason is that certain flows of the same plastic type from different product segments are treated together in one process, which means that the product flows from such processes are composed of plastics from different subsegments. Since the total flows of the individual plastic types are depicted in the Sankey diagram, the sum of the flows of the different segments was made and is specified in this particular sheet.

2 e.g. esters as functional group in polyesters (polymers)

3 e.g. olefins/alkenes as monomers of polyolefins (polymers), vinyls as monomers of polyvinyls (polymers)

4 thermoplastics, thermosets, elastomers

5 commodity/technical/specialty plastics

6 bio-/fossil-based

7 e.g. bio-degradable, compostable

8 The term “waste” as used in the present research work includes materials that are converted back to a useful product by recycling (see also [27]).

9 Note, however, that the share of secondary materials from pre-consumer waste in the total secondary materials is higher than the share of pre-consumer waste in the total waste, since more information is generally available on pre-consumer than on post-consumer waste, which facilitates recycling.
