# Supplementary material for: A high-resolution dataset on the plastic material flows in Switzerland
Source: Data Brief. 2022 Mar 2;41:108001. doi: 10.1016/j.dib.2022.108001 (PMC8914542; doi:10.1016/j.dib.2022.108001)
Supplement: Supplementary file 2 [file mmc2.pdf]

**Detailed Data Description Document**

# 1. Overview

This document provides a detailed description of the data acquision process as well as information on the collection, sorting and recycling processes in place. Information on the overall methodology, the system boundaries and the provided data pieces is available in the *Data in Brief* article.

This description is structured according to the sheets of the database, in which the flow data are stored according to the plastic life cycle (see Figure 1 of the *Data in Brief* article). Each of the Sections 2.1 to 2.12 refers to one sheet of the database, whereby the section names correspond to the names of the respective database sheets. The database sheet names are formatted in Calibri Light font, and the product subsegments are formatted in *italic*, when referred to in this description.

# 2. Data acquisition and background information

## 2.1. Imported plastics primary forms

Plastics used in product manufacturing in Switzerland can be imported in primary forms or as masterbatches. The definition of “primary forms” stems from the Federal Customs Administration [1] and refers to plastics in a liquid or pasty form, granulates, flakes or powder, or blocks of irregular shape, among others. The imported plastics in primary forms can be made from fossil, renewable or secondary resources (personal communication with a representative from Swiss-Impex [2], 2019-03-26). All plastics in primary forms registered under the tariff headings[1] 3901–3914 can contain additives [1]. The import amounts of the single plastic types were either directly available from Swiss-Impex [2] or estimated (see Section 2.5). Losses arising at polymer manufacturing abroad (which amount to 0.6% according to Kawecki et al. [3]) were not considered in the MFA.

Plastics imported as masterbatches constitute a non-negligible share of the total imported plastics for product manufacturing (personal communication with a representative from Swiss-Impex [2], 2019-03-26). Exemplary tariff headings under which masterbatches are imported include 3206.42[2], 3812[3] and 3824.99[4] (personal communication with a representative from Swiss-Impex [2], 2019-03-26, [2]). However, these tariff headings, besides masterbatches, also include other substances, which makes it difficult to accurately estimate the import of different polymer types as masterbatches. Thus, it was assumed that for each plastic type (except for PUR), an amount corresponding to 10% of the import as primary forms is additionally imported as masterbatches (see the related sheet in the database for further discussion on the estimation of masterbatch import and related plausibility check).

## 2.2. International trade parts and products

The import and export of plastics as parts and products was determined using the Swiss trade statistics provided in the Swiss-Impex database [2], by estimating the share of total plastics and individual plastic types in imported products. The considered Swiss-Impex tariff headings, which were assumed to contain plastics, are largely based on Kawecki et al. [3]; some additional headings were included, and some were allocated to different

---

[1] The trade categories under which items are imported are named tariff headings.

[2] Lithopone and other pigments and preparations based on zinc sulphide of a kind used to dye fabrics or produce colorant preparations (excl. preparations of heading 3207, 3208, 3209, 3210, 3212, 3213 and 3215)

[3] Prepared rubber accelerators; compound plasticisers for rubber or plastics, n.e.s.; anti-oxidising preparations and other compound stabilisers for rubber or plastics

[4] Chemical products and preparations of the chemical or allied industries, incl. those consisting of mixtures of natural products, n.e.s.

material flows from those in Kawecki et al. [3]. The applied shares of total plastics and individual plastic types for each tariff heading are specified in the database along with the related sources. The trade data were used for different purposes, as described in the related sections. The gathering and structuring of the data and corresponding calculations are described in Sections 2.2.1–2.2.8.

For all imported products, the amounts of plastics in primary forms required in product manufacturing abroad are higher than the amounts of plastics in the imported products themselves due to manufacturing losses. These manufacturing losses occur abroad and are not considered in the database, nor depicted in the Sankey diagrams (as they are for products manufactured in Switzerland).

### *2.2.1 Packaging*

The trade amounts of packaging products that are specifically listed as such in Swiss-Impex could be directly retrieved from that database. Additionally, packaging is traded along with packaged products. In trade statistics, the trade amounts are available for products including their packaging. Therefore, the amounts of (primary, secondary and tertiary) packaging which are traded along with the respective products were calculated using the method proposed by Kawecki et al. [3]. The tariff headings considered in the calculation were taken from Kawecki et al. [3]. The tariff headings that were not considered include live animals and plants, salt, sulfur, earths and stone, lime, cement, mineral materials, wood and articles thereof, cork, metals and articles thereof, and transportation (ships, railways, trams, aircraft, spacecraft). This method has its limitations, as some products traded under the excluded tariff headings may be packaged (e.g. silk, yarns, window frames from wood), and some products under the included tariff headings may not be packaged (e.g. chemicals delivered in tank trucks).

### *2.2.2 Building and Construction (B&C)*

The tariff headings relating to B&C were allocated to the B&C subsegments. Installation losses were only considered for those products allocated to B&C that are listed under "(Semi-)finished products into part manufacturing, product manufacturing, and use".

### *2.2.3 Automotive*

Regarding the international trade within the automotive segment, tariff headings relating to complete passenger vehicles and to vehicle parts were separately identified.

A considerable share of end-of-life (EoL) vehicles from Switzerland is exported. To determine which of the automotive exports in Swiss-Impex [2] concern not new, but EoL vehicles, the average export prices were compared to the average import prices of the relevant tariff headings. If the export price was smaller than one-third of the corresponding import price, then the exported products were considered EoL and treated as such in the

material flow analysis. This also implies that these respective amounts were not subtracted as exports from the total import for calculating the net import.

The same assessment of EoL status was conducted for automotive parts. If exported parts were considered EoL, it was assumed that they stem from a dismantling facility, i.e. from the sorting stage, and are exported partly for re-use and partly for recycling. However, EoL parts exported for recycling could also stem from replacement at a service or in case of an accident, which was not considered.

Regarding the compositions of automotive parts, average shares of total plastics and individual plastic types, referring to the automotive segment overall, were applied to each tariff heading of individual parts, except for the one of bumpers. For the tariff heading of bumpers (8708.10), specific shares of total plastics and plastic types were applied, due to their particular relevance for recycling today. All parts traded under the tariff heading relating to automotive parts (8708) were considered, as the share of total plastics applies to the whole vehicle including all parts. Under the tariff headings considered, not only parts for passenger vehicles are traded, but also parts for vehicles that are not included in the automotive segment in the model like trucks. However, as passenger vehicles constitute by far the largest share of traded vehicles, it was considered appropriate to use the amounts for passenger vehicles. Some vehicle parts are traded under different tariff headings, including some subheadings under 84 and 85 (such as 8512), 8706[5] and 8707[6]. These were not considered, which might lead to a slight underestimation of the amounts of imported plastics.

#### 2.2.4 Electrical and Electronic Equipment (EEE)

For the EEE segment, tariff headings referring to EEE products and EEE parts were distinguished from each other, as the individual amounts were necessary to calculate different material flows. The tariff headings referring to EEE with denominations including the word “parts”, as well as a few additional tariff headings clearly referring to parts of EEE products, were allocated to EEE parts.

To determine the amount of single plastic types used in EEE products, individual plastic shares were applied to each EEE subsegment. The allocation of the tariff headings from Swiss-Impex [2] to product subsegments was done using the first four digits of the tariff headings, with few exceptions (where the allocation was done on a more detailed level).

---

[5] Chassis fitted with engines, for tractors, motor vehicles for the transport of ten or more persons, motor cars and other motor vehicles principally designed for the transport of persons, motor vehicles for the transport of goods and special purpose motors of headings 8701 to 8705 (other than chassis fitted with engine and cab)

[6] Bodies, incl. cabs, for tractors, motor vehicles for the transport of ten or more persons, motor cars and other motor vehicles principally designed for the transport of persons, motor vehicles for the transport of goods and special purpose motor vehicle of headings 8701 to 8705

For EEE parts, the shares of total plastics and individual plastic types were applied to the total amounts of imported parts, not individual tariff headings, as the individual tariff headings were not allocated to subsegments. A few tariff headings containing mostly EEE (and considered as such in the database) also include products from other subsegments, e.g. vehicle electronics (see database sheet Product (sub)segments). Given the comparatively small amounts (the total net trade amount of vehicle electronics corresponds to about 0.4% of the net trade amount of automotive parts and 0.07% of the net trade amount of EEE), vehicle electronics were attributed to the *other EEE* subsegment instead of allocating the respective amounts to automotive parts. Similarly, some tariff headings of electrical and electronic (EE) medical equipment were allocated to *other EEE* instead of *medical and hygiene items*, as which EE medical equipment is accounted in the present research work.

For the EEE segment, the export of EoL products and parts is relevant (see Sections 2.8.4 and 2.10.4). The respective export amounts were determined analogously to the automotive segment (see Section 2.2.3).

### *2.2.5 Agriculture*

To the authors' knowledge, there is no specific tariff heading under which non-consumable agricultural products are exclusively listed. As such, it was assumed that agricultural products such as sheets and pipes are imported under the tariff headings listed under "(Semi-)finished products into part manufacturing, product manufacturing, and use" (see Section 2.2.8).

### *2.2.6 Household items, furniture, leisure and others*

Remaining tariff headings of products containing plastics are listed under Household items, furniture, leisure and others. They were allocated to the corresponding subsegments. There are few tariff headings that are not considered in this research work, although they may contain small amounts of plastics, such as clocks. Personal care products were neglected, since their relevance for recycling is negligible (their corresponding packaging, e.g. shampoo bottles, is included in the packaging segment).

### *2.2.7 Textiles*

The amounts of imported fibers and yarns as well as fabrics for textiles manufacturing, and textiles imported into use were taken from Kawecki et al. [3]. The amounts of PA (which was not considered by Kawecki et al. [3]) were calculated based on the share of PA used in textiles in proportion to the shares of the other plastic types.

### *2.2.8 (Semi-)finished products into part manufacturing, product manufacturing, and use*

Tariff headings listed under this header were allocated to the individual product segments. The further allocation to subsegments is described in Section 2.6, except for B&C, for which the allocation is directly specified on sheet Intl. trade parts and products. In addition to the knowledge on which products made from which plastic types are used in which segments, a number of other factors were also considered in the allocation to segments. Details on the assumed allocation shares can be found in the database. The amounts allocated to the agriculture segment were only used for comparison, and the actual imports were calculated as difference between the known total use amounts and the amounts of products manufactured in Switzerland. For semi-finished products, installation losses (e.g. cut-offs of pipes, insulation plates, sheets, etc.) were estimated as 10% of the import amounts, and they were subtracted from the total import amounts for determining the use amounts.

## 2.3. Manufacturing segments

Table 1 on the Manufacturing segments sheet ("Conversion of plastics in primary forms to products in CH") contains the amounts of plastics in primary forms that were converted to products in Switzerland, after subtracting the manufacturing losses. Table 2 ("OUT from Manufacturing CH total") includes, in addition to the amounts shown in Table 1, semi-finished parts imported for product manufacturing and assembling in Switzerland. The inclusion of semi-finished parts is only relevant in the automotive and EEE segments.

To determine the amounts of plastics converted to products in Switzerland (as provided in Table 1), the total inputs of each plastic type into manufacturing were calculated as a first step. As no virgin commodity plastics are produced in Switzerland [3], the total amounts of the individual commodity plastics converted to products in Switzerland were calculated as the sum of imports in primary forms [2] and imports as masterbatches (see Section 2.1), and secondary materials produced in Switzerland (see last paragraph of this section). Of the imported amount of PS, 25% were assumed to be XPS (which in the present research work is listed together with EPS). Of the manufacturing input amounts, the manufacturing losses (see next paragraph) were subtracted to get the plastic amounts in final products. The same procedure as for commodity plastics was applied for ABS and PC, which are neither produced in Switzerland according to the authors' knowledge. Polymer production of PA does take place in Switzerland. As respective exports are confidential, the total amount entering product manufacturing in Switzerland could not be derived from the trade statistics [2]. It was, therefore, calculated bottom-up, i.e. as the sum of PA used for product manufacturing in the different product segments (see Sections 2.3.1 – 2.3.7). Similarly, the HIPS and PUR product manufacturing amounts were also calculated using the bottom-up approach, as they were neither directly retrievable from

Swiss-Impex [2] (see sheet Imported plastics primary forms). The total amount of each plastic type converted into products was allocated to the individual product segments as described in Sections 2.3.1 – 2.3.7.

Manufacturing losses were estimated using a loss rate of 7% for non-textile product manufacturing and 10% for textile manufacturing [3] for all plastic types. The manufacturing losses, calculated via the loss rate, were subtracted from the total inputs of imported plastics and secondary material into manufacturing, where these input amounts were known. In contrast, for PA, HIPS and PUR, manufacturing losses did not need to be subtracted, as the converted amounts for the individual segments refer to final products (after production losses). Their production losses were calculated from the final product amounts and the loss rate. Installation losses such as cut-offs for insulation material or pipes produced in Switzerland were assumed to be included in the manufacturing losses.

The plastics used in product manufacturing are partly secondary plastics. For the imported secondary plastics, the respective amounts are already included in the amounts of plastics imported in primary forms (see Section 2.1). For the secondary plastics stemming from Switzerland, the respective amounts were added to the imported amounts of plastics in primary forms to get the total conversion amounts (before subtracting the manufacturing losses). The amounts of secondary materials from recycling in Switzerland resulting from the model partly depend on the amounts of plastic products manufactured in Switzerland. Therefore, two iterations were done to calculate the secondary material amounts, starting from not including any secondary materials from Switzerland in Swiss product manufacturing. The observed alternation of the secondary material amounts at the second iteration was minor, which is why this method is considered appropriate and no further iteration was done.

### *2.3.1 Packaging*

The plastics amounts converted to packaging products were calculated as difference between the total conversion amount of each plastic type (see the first part of Section 2.3) and the amounts used in manufacturing of products in other segments.

### *2.3.2 Building and Construction (B&C)*

For each plastic type, the amount used for product manufacturing in Switzerland was calculated as difference of the total use amount and the total import amount. The total use amount was calculated as the sum of the use amounts of the single subsegments (see Section 2.6).

### *2.3.3 Automotive*

No large-scale vehicle production takes place in Switzerland. The very small amount of vehicles produced in Switzerland was neglected. Automotive suppliers, however, produce

vehicle parts for original equipment manufacturers (OEMs) abroad. The case of automotive parts produced and used in Switzerland was neglected due to the absence of large OEMs in Switzerland (such parts might, however, be used as spare parts). Therefore, the manufacturing amount was derived from the trade statistics. The total amount of parts manufactured in Switzerland (Table 2 on sheet Manufacturing segments) was set equal to the gross export amount of (non-EoL) automotive parts. To get the converters' demand (Table 1 on sheet Manufacturing segments), the amount of imported semi-finished parts, assumed to be used for Swiss automotive parts manufacturing, was subtracted from the total amount of parts manufactured in Switzerland. An export surplus of semi-finished parts allocated to automotive means that the respective semi-finished parts are additionally made from plastics in primary forms in Switzerland and exported. In such case, the export surplus amount of semi-finished automotive products was added to the amount of exported automotive parts to get the total converters' demand, which in this case corresponds to the total amount of manufactured products.

#### *2.3.4 Electrical and Electronic Equipment (EEE)*

The total amount of plastics in EEE produced in Switzerland (Table 2 on sheet Manufacturing segments) was calculated as the sum of plastics in primary forms converted to EEE and plastics in imported EEE parts assembled in Switzerland.

The amount of plastics in primary forms converted to EEE was calculated using the share corresponding to EEE of the total amount of plastics in primary forms converted to products in Switzerland [4].[7] That amount was multiplied with the share corresponding to the considered plastics, since the conversion amount used for the calculation [4] includes all plastics. From the resulting amount, the manufacturing losses were subtracted.

Additionally, OEMs import parts for assembly in Switzerland. The respective amounts listed under the tariff headings referring to EEE parts, as well as semi-finished products allocated to EEE, were added to the total conversion amount.[8] If there is a net export of semi-finished products, the respective amount is included in the converters' demand and was thus not added.

The total manufacturing amounts of the individual plastic types were calculated as the differences between the use and net import amounts (Section 2.6).

---

[7] The share corresponding to EEE manufacturing available from Röschli [4] had been determined considering all related companies (personal communication with Röschli, 2019-04-18).

[8] Parts imported for product manufacturing could also be declared under tariff headings which are in the present model allocated to EEE products (not EEE parts), which means the respective amounts were not considered for calculating the total amount of EEE produced in Switzerland. If that is the case, a respective consideration would lead to a higher amount of products manufactured in Switzerland. The allocation necessary therefor was, however, considered difficult, and possible additional parts used for product manufacturing in Switzerland were neglected.

The conversion amounts (after conversion losses) for the individual plastic types were calculated by subtracting the amounts of imported parts and semi-finished products from the total production amount. If a net export of semi-finished parts occurs, the respective amount needs to be additionally produced. The amount is in such case included in the converters' demand.

For comparison, the total amount of plastics used to produce EEE in Switzerland was calculated in an alternative way. The alternative calculation was based on the export rate of the Swiss mechanical and electrical engineering industries (MEM industries) (79% in 2017 based on Swissmem [5]) and the gross export amount (from sheet Intl. trade parts and products). This assumes that the export rate for the EEE subsector is similar to the overall export rate of the MEM industries. The total export amount of EEE was calculated as the sum of the exports of parts (declared as such) and products (semi-finished products allocated to EEE were neglected). This calculation resulted in a higher amount of Swiss produced EEE (about 1.5 times as high as in the present model – see sheet Intl. trade parts and products), which is likely due to the fact that not all exported products are actually produced in Switzerland, but rather only traded via Switzerland. Therefore, the applied calculation method is considered more accurate.

#### *2.3.5 Agriculture*

The total amount of agricultural plastic products manufactured in Switzerland was calculated using the respective share in the total converters' demand according to Röschli [4], multiplied with the share corresponding to the total considered plastic types. The total amount was broken down into the amounts of the individual plastic types according to the shares of the plastic types in the use phase (based on Kalberer et al. [6], see Section 2.6). The total amount of agricultural products produced in Switzerland is very small compared to the import amounts [6]. The possible inaccuracies due to using the same shares as for the use phase is therefore considered irrelevant.

#### *2.3.6 Household items, furniture, leisure and others*

For the product segment Household items, furniture, leisure and others, the calculations differ depending on the plastic type. For the commodity plastics, the amounts used in product manufacturing in Switzerland for the individual subsegments were calculated based on the total Swiss converters' demand and the shares used in the Household items, furniture, leisure and others subsegments [4], including assumptions. Manufacturing losses (see Section 2.3) were subtracted.

The amounts of PC and ABS were calculated as the difference between the total imported amounts of these plastic types in primary forms and the amounts already allocated to the other segments (automotive and EEE). For HIPS, PA and PUR, the amounts for manufacturing in Switzerland were calculated by subtracting the net import of products from

the total use amounts (see Section 2.5). This estimation was necessary, since the net import amount of plastics in primary forms could for these plastic types not be directly retrieved from the import statistics.

#### *2.3.7 Textiles*

No fiber manufacturing takes place in Switzerland (personal communication with Röschli, 2019-04-18). The amounts of individual plastic types in total textiles manufacturing in Switzerland were thus modelled to correspond to the amounts of imported fibers/yarns and fabrics (from the sheet Intl. trade parts and products).

### 2.4. Manufacturing subsegments

#### *2.4.1 Packaging*

The shares of plastic types in individual packaging subsegments in the total packaging manufacturing amount were unknown. For each plastic type, the same distribution to the subsegments as for the total use amount was assumed for product manufacturing in Switzerland.

#### *2.4.2 Building and Construction (B&C)*

The B&C product manufacturing amounts for Switzerland were calculated as the difference between the use and net import amounts.

#### *2.4.3 Automotive*

The automotive segment is not further divided into subsegments.

#### *2.4.4 Electrical and Electronic Equipment (EEE)*

The Swiss manufacturing amounts for the plastic types in the individual subsegments were calculated as the difference between the use and net import amounts.

#### *2.4.5 Agriculture*

The total amount for product manufacturing in Switzerland for each plastic type was calculated as described in Section 2.3.5. The amounts were allocated to the subsegments by assuming that the subsegment shares are the same as for the use phase (the distribution of the total manufacturing amount among the subsegments was not considered relevant, since the share of products manufactured in Switzerland of the total use is negligible).

#### *2.4.6 Household items, furniture, leisure and others*

For the commodity plastics, the amounts for manufacturing in Switzerland for the Household items, furniture, leisure and others subsegments were calculated based on the total Swiss plastics converters' demand, the shares of the respective subsegments from Röschli [4], and the share of each plastic type used in each subsegment based on the shares from sheet Plastic types. *Toys* and *sports items* were not separately specified in Röschli [4]; they were assumed to amount to 10% each in the category "other products" of Röschli [4]. The manufacturing losses were subtracted. For the technical plastics and PUR, the total manufacturing amounts (after manufacturing losses) for these plastic types from the sheet Manufacturing segments were used as the calculation basis. The respective amount for each plastic type was distributed to the subsegments based on the share of the single subsegments in the converters' demand (the share referring to the total plastics amount of the subsegment) and the share of the respective plastic type out of all plastics used in each subsegment (see sheet Manufacturing subsegments of the database for the exact calculation procedure). The latter was assumed based on the sheet Plastic types.

#### 2.4.7 Textiles

The Swiss manufacturing amount for agrotextiles was calculated as the difference between use and net import. For the subsegments other than agrotextiles, the total amount of each plastic type used in textiles manufacturing (available from sheet Manufacturing segments), minus the amount used for agrotextiles manufacturing, was equally distributed among the subsegments. This was considered reasonable, since the total amount of textiles manufactured in Switzerland is small compared to the import amount.

### 2.5. Use segments

The data on the sheet Use segments are structured into products manufactured in Switzerland («Manufacturing CH"), imported products ("Import (net)") and Swiss consumption amounts ("Use CH"). The use amounts, listed under "Use CH", were calculated as the sum of the amounts of products manufactured in Switzerland (listed under "Manufacturing CH") and the net import amounts (listed under "Import (net)") for the packaging, B&C, automotive and textiles segments for all plastic types. The same calculation method was used for the plastic types other than HIPS, PA and PUR in the Household items, furniture, leisure and others segment; for HIPS, PA and PUR, the total use amounts were estimated based on the ratio of the respective plastic types to the commodity plastics used in this segment, as their amounts for manufacturing in Switzerland were not known (see Section 2.3). For the EEE and agriculture segments, the calculations are described under Sections 2.2 and 2.6, respectively.

The amounts listed under "Manufacturing CH" used in calculating the use amounts refer to product manufacturing in Switzerland, involving the conversion of plastics in primary

forms to products and the assembly of imported parts in case a product is assembled in Switzerland by an OEM (relevant for the automotive and EEE segments).

The amounts listed under “Import (net)” refer to the net import for use and were derived from the sheets Intl. trade parts and products and Use subsegments. For B&C, they refer to the amounts of installed products. If considered relevant, respective installation losses were subtracted from the import amounts (see Section 2.2). For the automotive segment, the net import amount equals to the sum of net imported vehicles and imported spare parts (the gross import amount of automotive parts was considered to correspond to spare parts) minus the exports of parts produced in Switzerland (corresponding to the gross export of parts, see Section 2.3.3). Of the imported vehicles, only the share of non-textile plastics was considered, since *mobility textiles* contained in vehicles were allocated to textiles. Additionally, in case there was an export of semi-finished parts manufactured in Switzerland, the respective amount was subtracted. If there was a net import of semi-finished parts, they were not added to the import into the use phase, because it was assumed that they are used for parts manufacturing in Switzerland (see Section 2.3.3) and are therefore included in the respective amounts. Using the described calculation method, the complete amount of automotive parts and semi-finished parts manufactured in Switzerland was modelled to be exported (see Section 2.3). Since more vehicles and spare parts were imported than vehicle parts produced in Switzerland exported, overall there is a net import of plastics. For EEE, the imported (semi-finished) parts used for product manufacturing are included in the amounts for total manufacturing in Switzerland (see Section 2.3), and only the imported products and the net exported semi-finished products (if this is the case for a specific plastic type) are listed under “Import (net)”.

The resulting use shares of the individual product segments in Switzerland are well in line with the respective shares in Europe [7]. The absolute segment sizes are very close to the results from the previous Swiss studies [3], [8]. This is plausible, as the plastic consumption in Switzerland is assumed to have remained rather constant over the last years based on the import development under the Swiss-Impex tariff heading 39 – “Plastics and articles thereof”. The tariff heading 39 as an indicator is considered suitable, as it includes plastic products and plastics in primary forms used for product manufacturing in Switzerland.

### 2.6. Use subsegments

On the sheet Use subsegments of the database, the net import into use (listed under “Import (net)”) and the use amount (listed under “Use CH”) are provided for each plastic type in each subsegment.

The net import amounts for the B&C, automotive, EEE, Household items, furniture, leisure and others and textiles subsegments are the respective sum of the import amounts of relevant tariff headings in the trade statistics (provided in the sheet Intl. trade parts and

products, see Section 2.2 for details). The allocation of tariff headings to the individual subsegment(s) is specified in the sheet Intl. trade parts and products. For packaging and agriculture, the import amounts were calculated as the difference between the use amounts and the Swiss manufacturing amounts (see Sections 2.6.1 and 2.6.7 for more details).

### *2.6.1 Packaging*

The total packaging use amount of each plastic type (see Section 2.5) was allocated to the individual subsegments based on the reported shares of plastic types in different packaging items used in the United Kingdom [9] with some adaptions, as specified in the sheet Packaging raw data & calc. For consumer packaging, the same plastic type composition was assumed for food and non-food consumer packaging items. One exception is bottles from HDPE and PET, for which different shares for food and non-food bottles were used based on WRAP [9]. According to the authors' knowledge, flexible food packaging consists of a much higher share of multi-layer materials compared to flexible consumer non-food packaging (also see [10]). The shares of the individual commodity plastics, however, might still be similar for food and non-food, thus the described approach is considered sensible.

The net import was calculated as the difference between the use and manufacturing amounts in Switzerland. As the same shares as for the use amounts were assumed for the distribution of the total amount of each plastic type in packaging manufactured in Switzerland to the individual packaging subsegments (see Section 2.4), the same composition for use, manufacturing and import resulted.

### *2.6.2 Building and Construction (B&C)*

The plastic-type-specific use amounts were calculated bottom-up for the subsegments *pipes and ducts*, *flooring*, *window profiles* and *roof lining*, by multiplying B&C statistics on floor area or pipe network length ([11]–[13]) with data on the specific use of the individual plastic types in the above-mentioned applications. Details on the calculation procedure can be found in the sheet B&C bottom-up. For the subsegment *thermal insulation*, data for the considered plastic types was available from Wiprächtiger et al. [14]; the use amounts of EPS and XPS available for the year 2015 were scaled to the year 2017 (see database for details). The total use amount for the *other B&C products* subsegment was estimated based on its size ratio to the other B&C subsegments from APME [15], and was broken down into individual plastic types based on the average shares of individual plastic types used in B&C from PlasticsEurope [7].

No window profiles are currently produced in Switzerland (personal communication with Steinauer, 2018-12-05). Accordingly, the shares allocated to *Window profiles* of the tariff

headings under which windows are imported were chosen so that the import amount corresponds to the use amount.

### 2.6.3 Automotive

The automotive segment is not further divided into subsegments.

### 2.6.4 Electrical and Electronic Equipment (EEE)

The total use amount was calculated as the sum of the total amount of EEE manufactured in Switzerland and the total imported amount. The total use amount was allocated to the subsegments and plastic types based on shares compiled from different sources ([7], [16]–[19], see the sheet variable parameters of the database).

The net exports of semi-finished products were allocated to *other EEE* due to a lack of a more specific category and since they constitute only a small share of the total trade amount of EEE. If there was a net import of semi-finished products, they were assumed to be used for manufacturing in Switzerland and included in the manufactured products. Note that the net import is provided in the sheet Use subsegments. The gross import is higher than the net import, so the ratio of imported EEE to the amount of EEE produced in Switzerland seems plausible.

### 2.6.5 Agriculture

The use amounts of the different plastic types in the subsegments were known from Kalberer et al. [6], and match well with the total use amounts in *agriculture* from Kawecki et al. [3].

The import of agricultural products was not directly available from the trade statistics (see Section 2.2) and was calculated as the difference between the use amounts and the amounts of products manufactured in Switzerland. The estimated overall net import constitutes the vast majority of the use amount, which was also found by a related study [6].

### 2.6.6 Household items, furniture, leisure and others

The use amounts were calculated as the sum of the Swiss manufacturing and the net import amounts. The imported amount of semi-finished products of each plastic type was equally allocated across the Household items, furniture, leisure and others subsegments. The Swiss-Impex tariff headings with the biggest contributions to the total net import amount were the ones relating to mattresses and other bedding articles (tariff heading 9404), articles of plastics not elsewhere specified (3926.90), motor vehicles for the transport of goods (8704), and articles for physical exercise (9506).

### 2.6.7 Textiles

The use amounts of textiles (excluding agrotextiles) were calculated as the sum of Swiss product manufacturing and net import.

For agrotextiles, the use amounts were available from Kalberer et al. [6] and in line with Kawecki et al. [3]. To determine the import amount, the share allocated to agrotextiles of the tariff headings under which agrotextiles are imported was chosen so that the largest share of the use amount is imported, as it is known to be the case from Kalberer et al. [6].

*Mobility textiles* are imported as part of imported passenger vehicles. The amount of mobility textiles contained in the imported vehicles was calculated as a share of all plastics contained in passenger vehicles. *Mobility textiles* that may be contained in separately imported automotive parts were neglected. The total amount was broken down on the plastic type level based on the respective shares in the sheet Plastic types.

## 2.7. Collection segments

The collection amounts for each segment are compiled in this sheet from the sheet Collection subsegments. For further information, see Section 2.8.

## 2.8. Collection subsegments

In the sheet Collection subsegments, all outflows from the use stage as well as the inflows and outflows of the separate collection stage are provided. The flow amounts are structured according to the life cycle stages that they connect (underlined subheadings in Sections 2.8.1–2.8.7). The amounts listed under "OUT from Use" refer to the waste amounts. For all existing collection systems, the collection amounts are specified separately (under "OUT from use – IN to separate collection"). "OUT from Use – IN to energy recovery in WTE plant" refers to the waste collected for thermal treatment in WTE plants[9]. Besides, certain wastes are exported, or their destiny is unknown. From the separate collection, the material goes to sorting in Switzerland or abroad, or, in few cases, to thermal treatment in cement kilns or WTE plants (as listed under "OUT from separate collection").

### *2.8.1 Packaging*

OUT from Use:

For packaging, the waste amounts correspond to the use amounts, since most packaging products have a lifetime of less than one year. Longer-lasting products (such as re-usable food containers, buckets or other products listed under, e.g., the tariff heading 3923.10) were neglected as they constitute a small share of the total packaging amount.

---

[9] The respective waste collection takes place via bag collection systems, skips, delivery to recycling centers or direct delivery to WTE plants.

OUT from use – IN to energy recovery in waste-to-energy (WTE) plant:

The amounts given in the database refer to the residual waste collection that is subject to incineration. Any material that was possibly disposed of via hazardous waste incineration plants, mainly relevant for *non-consumer packaging C&I – manufacturing* waste, was not separately quantified and is included in the amounts specified as going to WTE plants.

OUT from use – IN to separate collection:

The existing packaging waste collection systems were divided into consumer (C) and non-consumer (NC) systems, based on the origin of the waste that they mainly target – private consumers or companies (independently of their size), respectively. The exception is the PET drinking bottle collection, which is considered as a consumer system regardless of the waste origin (*non-consumer packaging C&I - hospitality – bottles* are as well collected via this collection). An overview on the collection systems is provided in Table 1. The procedure for determining the collection amounts is described below in the subsections relating to the single collection systems (Sections 2.8.1-A–2.8.1-I). How each collection is further processed ("OUT from separate collection") is also described in these subsections.

The share of the packaging waste that falls under municipal solid waste includes all packaging subsegments related to consumer waste and waste from companies with <250 full-time employees in non-consumer packaging subsegments [20]. Besides, municipal solid waste includes waste from subsegments of other segments related to consumer waste, such as *toys*.

*Table 3: Overview of existing collection systems for (consumer and non-consumer) packaging in Switzerland. B&C: building and construction; C&I: commercial and industrial.*

| ***Collection system*** | *Responsible organization / stakeholder* | *Target items / collected item* | *Explicitly excluded items* | *Mis-collected items* | *Data sources* |
|---|---|---|---|---|---|
| **2.8.1-A PET drinking bottle collection** | association Verein PRS PET-Recycling Schweiz | PET drinking bottles with PE or PP caps from consumers and hospitality | other food and non-food bottles, food PTTs, PE shrink film, other waste | PE and PP bottles (completely allocated to food bottles), LDPE foils and items from other materials (aluminum cans and lids, beverage cartons) | [21] (data for 2018); personal communication Würmli, 2019-07-09; [22]; |
| **2.8.1-B Hollow bodies collection** | retailers (Migros, Coop, ALDI SUISSE, Lidl Schweiz) | food bottles and consumer non-food bottles (mainly from HDPE, PP, PET), | PET drinking bottles, consumer non-food bottles from garden / do-it-yourself / automotive applications, PTTs, films, | PET drinking bottles, food and non-food PTTs of PET, PP and PS, beverage cartons | [23] (data for 2016); [24]; [25]; [26]; [27]; [28]; |

| | | | | | |
|---|---|---|---|---|---|
| | | beverage cartons[10] | tubes, beverage cartons[11] | | |
| **2.8.1-C Mixed-plastic collection bags / points** | around 10 different systems | food and consumer non-food bottles / PTTs / films / bags, non-consumer shrink and stretch films / bottles / PTTs, beverage cartons, office materials, garden furniture, baskets, flower pots, boxes, pipes[12], toys, garden toys, balls (exc. PVC), composite packaging[13] | PET drinking bottles, toys, garden hoses, disposable tableware, plastics in combination with other materials, silo wrapping foils, beverage cartons, PVC items, EPS, electric cables, cable conduits[14] | | [23]; [29]; [30]; [31]; [32]; [33]; |
| **2.8.1-D EPS collection bags** | EPS Verband Schweiz | EPS ("Styropor", "Sagex" = PS rigid foam) packaging and pre-consumer insulation material (e.g. cut-offs) | fruit and meat packaging (from PS rigid foam), loose fills, other plastics | | [44]; [45]; personal communication Meier, 2019-10-28; [36]; [37]; personal communication Dalla Bona, 2020-10-30; |
| **2.8.1-E B&C plastics collection bags** | Verband des Schweizerischen Baumaterial-Handels (VSBH) (respective collection was in 2018 taken over by Eurobaustoff (German company with | films / bags from PE (e.g. shrink films, packaging, e.g. of bricks on pallets, insulation material, windows, construction films ("Baufolien"), cover sheeting) | pipes | | [38]; personal communication Wyder, 2019-07-12 (data for 2017); [39]; personal communication Küchler, |

[10] Beverage cartons were only collected by one retailer.

[11] While beverage cartons are a target collection item for one system, they are excluded from the collection for other systems.

[12] Pipes listed as target items for one system were neglected in the calculations (for the other two considered systems (see column *Data sources*) pipes were not listed under target items), the complete collection amount was considered as consumer products.

[13] These collection systems are listed under consumer packaging collection systems, as most target items are consumer packaging products. However, also non-consumer and non-packaging products are target items of some systems.

[14] Some products are target items for certain systems and non-target items for other systems.

| | | | | |
|---|---|---|---|---|
| | branch in Switzerland)), Handelsgenossenschaft des Schweizerischen Baumeisterverbandes HG Commerciale (HGC) | | | 2019-07-17; personal communication Tonner, 2019-07-16; |
| **2.8.1-G C&I collection systems - Manufacturing** | disposal companies[15] | LDPE shrink films (pallet wrapping films of natural color without print), colored and multi-layer films[16], HDPE boxes (assumed also PP), canister, PE buckets with lids, additional items based on contract, production waste and items of other than the considered materials | | personal communication Huhn, 2019-06-20; personal communication Stotz, 2019-05-15; personal communication Tonner, 2018-12-06 and 2019-04-09; [40]; |
| **2.8.1-H C&I collection systems - Retail** | Migros, Coop, Volg, Denner, ALDI SUISSE, Lidl Schweiz; collection via disposal companies, Senn Brunnen collection bag system mainly used by smaller retailers | LDPE stretch films (almost exclusively; used as tertiary packaging, if not reusable containers are used; few PP films), few other plastics (unspecified) | | personal communication Stotz, 2019-05-15 and 2019-07-10; [40]; personal communication Senn, 2019-05-23 and 2018-07-15; Senn Brunnen (2020); personal communication Tonner, 2019-07-16; personal communication Huhn, 2019-06-20; [9]; |

[15] companies contracted for taking care of the disposal and recycling of waste for their customers

[16] assumed to be collected, as similar pre-consumer waste is collected for energetic use in cement kilns

| | | | | |
|---|---|---|---|---|
| **2.8.1-I C&I collection systems - Hospitality** | disposal companies, caterings, restaurants, hospitals | shrink film, PP buckets, containers of non-hazardous cleaning agents, reusable boxes for food and bottled beverages, PE tanks | | personal communication D. Bachmann, 2019-07-10; personal communication Nadler, 2019-07-17; [40]; |

### 2.8.1-A. PET drinking bottle collection

<u>OUT from use – IN to separate collection:</u>

The total collection amount of the considered plastics was calculated by subtracting the moisture content and the mis-collected items made of not considered materials from the total collection amount. The moisture content was assumed to amount to a quarter of the moisture content of the hollow bodies collection (Section 2.8.1-B), the underlying rationale is provided in the database sheet variable parameters. The amount of mis-collected items made of not considered plastics was assumed based on Verein PET-Recycling Schweiz [21] and personal communication with Würmli, 2019-07-09.

The collection amount constituted by PET, HDPE, and PP in PET drinking bottles was calculated by subtracting the amounts of mis-collected HDPE, PP and LDPE from the total collection amount constituted by the considered plastics. The mis-collected amounts were calculated from the amounts of the respective plastics sorted out ([21], personal communication with Würmli, 2019-07-09) by considering the sorting losses. The resulting amount was then attributed to PET, HDPE, or PP based on the secondary material shares calculated from Verein PET-Recycling Schweiz [21]. The share of bottle caps constituted by HDPE and PP, respectively, was available from personal communication with Würmli, 2019-07-09. To calculate the total collected HDPE and PP amounts in food bottles, the respective mis-collected amounts[17] (constituted by e.g. milk bottles) were added to the amounts of HDPE and PP from bottle caps. The mis-collected LDPE amounts fall under *consumer non-food bags*.

To allocate the total amount of the collected PET drinking bottles to *food bottles* and *non-consumer packaging C&I - hospitality - bottles*, an equal collection rate of PET drinking bottles from both subsegments was assumed. The collected amount of PET in drinking bottles form hospitality was calculated from the overall collection rate of PET drinking bottles including caps (see the sheet variable parameters) and the use amount of PET in

[17] allocated to *food bottles*; could, however, as well include *consumer non-food bottles*

*non-consumer packaging C&I - hospitality - bottles*. The corresponding amounts of collected HDPE and PP caps from *non-consumer packaging C&I - hospitality - bottles* were calculated based on the shares of secondary materials from bottles respectively caps [21]. The HDPE / PP shares of the caps were available from personal communication with Würmli, 2019-07-09. The amounts allocated to *food bottles* were calculated by subtracting the collected PET, HDPE, and PP amounts in *non-consumer packaging C&I - hospitality - bottles* from the total amounts collected via the PET drinking bottle collection for each of these plastic types (calculated as described in the previous paragraph).

<u>OUT from separate collection - IN to sorting CH:</u>

Based on Haupt et al. [41], a small share of about 5% of the collection was exported in 2012 for sorting, which was expected to be lower in 2017 due to current market dynamics. The minor export amount was, therefore, neglected, and the complete collection was modelled to be sorted in Switzerland.

#### 2.8.1-B. Hollow bodies[18] collection

<u>OUT from use – IN to separate collection</u>

The gross hollow bodies collection amount and moisture content (11%) were available from Dinkel et al. [23]. It was assumed that 10% of the total collection amount could be attributed to mis-collected beverage cartons (including the contained plastic films, which were not recycled and were thus neglected) and other mis-collected materials. This mis-collection amount was subtracted, additionally to the moisture, from the gross collection amount to get the net collected amount of considered materials. The total collection amount was allocated to subsegments and plastic types based on personal communication with Tonner, 2019-04-09[19].

<u>OUT from separate collection - IN to sorting CH / sorting abroad:</u>

About 3000 t of the hollow bodies collection were sent to a recycling plant in Switzerland in 2017. Of this, one-third was exported for near-infrared (NIR) sorting, one-third was NIR-sorted in Switzerland, and the last one-third underwent float-sink sorting at the recycling plant itself (personal communication with Tonner, 2019-04-09). It was assumed that the remaining collection amount (1322 t) was sorted in Switzerland by using NIR technology by the companies Müller Recycling, Papirec and RC-Plast (based on personal communication with Tonner, 2019-04-09, personal communication with Geisselhardt, 2019-11-29, [58–60]).

---

[18] The denomination “hollow bodies” only refers to bottles for this collection scheme.

[19] The respective information regarding the composition of the collection is based on one plant. Still, it was considered to be representative for the whole collection, because the predominant part of the total hollow bodies collection is sorted at the respective plant.

### 2.8.1-C. Mixed-plastic collection bags / points

OUT from use – IN to separate collection

This section refers to two similar collection systems, collecting mixed plastics via bags or at collection points. According to Dinkel et al. [23], the total amount of plastics collected via the hollow bodies, as well as mixed-plastic collection bags and points systems amounted to 18'000 t. From that amount, 11% moisture content were subtracted Dinkel et al. [23]. By subtracting the net hollow bodies collection amount (see above), the total amount collected via mixed-plastic collection points and bags was gained. Collected items made from other materials than the considered plastic types were subsequently subtracted based on the average composition of one of the bag collection systems [30]. The allocation of the remaining amount to mixed-plastic collection bags and points, respectively, was done based on the total collection amounts of these two systems [23], assuming that both collections contain a similar share of moisture and non-target items. Products belonging to other subsegments (*household items*, *toys*) that are also collected via mixed-plastic collection bags, are listed under the Household items, furniture, leisure and others segment and were subtracted from the total here.

The allocation to subsegments was done based on detailed compositional data available for one bag collection system [30]. A similar composition was assumed for collection points. However, the plastic-type composition of the collected products was not assessed in Gasser et al. [30], neither does the composition data distinguish whether a collected product comes from a food or a consumer non-food application. Therefore, the same collection rate was assumed for food and consumer non-food items, as well as for all plastic types used in the collected products. Thereby, the amounts of collected EPS and PVC, which are partly explicitly excluded from the target items, are probably overestimated, even if part of these plastics might still have been mis-collected.

OUT from separate collection - IN to sorting abroad:

The mixed-plastic collections were modelled to be completely exported for sorting because of a lack of sorting infrastructure in Switzerland for mixed consumer packaging waste[20] (personal communication with Tonner, 2019-04-09, personal communication with Geisselhardt, 2019-11-29).

### 2.8.1-D. EPS collection bags

OUT from use – IN to separate collection

---

[20] Swiss sorting plants stating that they are able to sort out films [44], or that accept mixed plastics waste from collection points [43] were assumed not to fulfill this function.

It was not possible to obtain exact information on the collected amounts of EPS. According to personal communication with different related stakeholders, only small amounts of post-consumer waste are currently being collected. Based on this, it was assumed that 10% of the waste from the target subsegments (*food other*, *consumer non-food PTTs*, and *consumer non-food other*) are collected. Collection points exist in numerous municipalities, recycling centers, and construction material sales points.

<u>OUT from separate collection - IN to sorting CH:</u>

The complete collection is sorted in Switzerland (personal communication with Meier, 2019-10-28).

#### 2.8.1-E. B&C plastics collection bags

<u>OUT from use – IN to separate collection</u>

The collected amounts were calculated based on data from the two collection operators on the sales of collection bags, and their respective volumes and weights. This calculation resulted in a total collection amount of approximately 200 t per year. At the same time, it was known that in Switzerland, 250–300 t per year of films from the two collection operators are recycled. Thus, 250 t per year were assumed to be the total collected amount. Although cover sheeting does not fall under packaging, it is one of the target collection items and is thus included in the collection amounts.

<u>OUT from separate collection - IN to sorting CH:</u>

The total collection amount was modelled to be sorted in Switzerland, as the estimated collection amount roughly corresponded to the amount of materials that were sorted and recycled in Switzerland (personal communication with Tonner, 2019-07-16).[21]

#### 2.8.1-F. Agricultural packaging collection systems

<u>OUT from use – IN to separate collection</u>

Two agricultural waste collection systems were the basis for the model ([45], [46]). One of these almost exclusively (personal communication with Tonner, 2019-04-09), and the other exclusively collects silo wrapping films (personal communication with Senn, 2018-07-15). The Re-Log system (another collection system, also targeting agricultural waste other than silo wrapping foils, see [46]) is almost not existent anymore according to per-

---

[21] This also fits with the information that the collection by HGC is completely sorted in Switzerland at InnoRecycling and Plastic-Ti Recycling (personal communication Küchler, 2019-07-17), and that at InnoRecycling also material collected by VSBH/Eurobaustoff is sorted (personal communication Tonner, 2019-07-16).

sonal communication with Senn, 2019-05-23. Possibly, some farmers delivered their plastic waste to recycling centers, but no such information was obtained. Therefore, the collection amounts of agricultural packaging were assumed to be zero in the model.

#### 2.8.1-G. Commercial and industrial (C&I) collection systems – manufacturing packaging

OUT from use – IN to separate collection

Disposal companies, contracted for taking care of the disposal and recycling of waste for their customers, collect waste from companies. However, based on personal communication,[22] separated waste collection from companies was rare, especially as the cost for plastic recycling can be higher than that for WTE incineration (personal communication with A. Schaub, 2018-12-04; personal communication with Burkhalter, 2018-12-06 and 2018-12-10; personal communication with Senn, 2019-05-23; personal communication with Kaufmann, 2019-09-13). As such, it was assumed that 10% of the waste amounts of the target collection items known from a disposal company (personal communication with Huhn, 2019-06-20) were separately collected. It was assumed that PP boxes and buckets, which were conveyed to recycling by disposal companies for hospitality stakeholders, also belonged to the collected items. Only for rigid packaging made from HDPE, the collection amount was estimated in a different way: the respective waste amount going to sorting and recycling in Switzerland was known. It was assumed that twice that amount was collected, i.e., the same amounts were recycled abroad and in Switzerland.

OUT from separate collection – IN to sorting CH / sorting abroad / energy recovery in cement kiln:

Of the total collection amount, the amounts sorted in Switzerland were known (personal communication with Tonner, 2018-12-06 and 2019-04-09, [40], [47], including assumptions – see sheet mat. flows of a sort&rec plant). Of the remaining collection amount, 10% were assumed to be used as substitute fuel in cement kilns, and the remainder sorted abroad. The assumption that, besides the waste targeted for recycling, a small share of the collection is used in cement kilns, is based on the following information: Certain collected wastes like printed or multi-layer films are not suitable for recycling. This can regard collected items made from the considered plastic types that were not explicitly mentioned in Table 3 under "target items" (personal communication with Huhn, 2019-06-20). Financial aspects can also play a role. For disposal in WTE plants, the cost is generally higher

[22] From one big retailer, the information was obtained that in their manufacturing subsidiaries, plastics were partly collected in a mixed plastic collection, but generally they were not separately collected (personal communication with Stotz, 2019-05-15). From a large company working with hazardous chemicals, the information was obtained that these chemicals were delivered to the company in plastic containers, and that the same plastic containers were used for the transportation of waste chemicals to the WTE plant or hazardous waste incineration plant and incinerated along with them.

than that for disposal in cement kilns; thus, it was assumed that disposal companies did not send separately collected plastic waste to WTE plants (only waste that they directly collected in the burnable fraction, which is not regarded as separate collection in the model). Regarding sorting abroad, it was mentioned by a disposal company that their collected films were completely recycled abroad, while the other target items explicitly mentioned above were partly recycled in Switzerland, partly abroad (no such items went to cement kilns) (personal communication with Huhn, 2019-06-20).

#### 2.8.1-H. C&I collection systems – retail packaging

<u>OUT from use – IN to separate collection</u>

For *non-consumer packaging C&I - retail - films*, two estimates were made for the total collected amount. One estimate was done by summing up the known separately collected waste amounts by two big retailers, as well as the additional waste amounts from other retailers that were collected via a bag collection system (and possibly also via disposal companies). The latter were estimated based on the roughly known total amount of *non-consumer packaging C&I - retail - films* waste undergoing sorting and recycling in Switzerland, assuming that half of the total collection amount was recycled in Switzerland and half abroad (see sheet NC packaging raw data for details). The second estimate was done by scaling up the known amount that was separately collected by two retailers based on their market shares (see sheet NC packaging raw data). However, smaller retailers, due to a possibly more difficult logistic situation, did probably not collect a share of tertiary packaging as high as the big retailers did, although collection systems existed also for smaller retailers. Therefore, the average of the first estimate and the second estimate (being by one-third higher than the first estimate) was used as the final total collection amount in the model.

Based on information from [40], the separate collection was partly even more specific than films (e.g. transparent / colored, LDPE / PP films were separately collected). The total amount of collected PP films from retail was calculated based on the data from one retailer by scaling up the known amount of PP films collected by that retailer, using the same ratio of the total amount of films collected by that retailer to the total amount of all collected films. The rest of the collected films was attributed to LDPE.

For *non-consumer packaging C&I - retail - other*, the total amount that was separately collected was calculated by scaling up the available amount from one retailer via its market share. The obtained amount was equally split between PET and PP based on [9].

<u>OUT from separate collection - IN to sorting CH / sorting abroad / energy recovery in cement kiln:</u>

The collected LDPE films were partly recycled in Switzerland (personal communication with Tonner, 2018-12-06 and 2019-04-09, [40], including assumptions – see sheet mat.

flows of a sort&rec plant for detailed information and amounts; personal communication with Senn, 2019-05-23 and 2018-07-15). They stemmed from the big retailers, from collection bag systems for smaller retailers, and from disposal companies (personal communication with Tonner, 2019-07-16, personal communication with Senn, 2019-05-23 and 2018-07-15). Analogously to manufacturing packaging (see Section 2.8.1-G), it was assumed that 10% and 90% of the film amount not conveyed to recycling in Switzerland were used as alternative fuel in cement kilns[23] and recycled abroad, respectively. The latter was further supported by a retailer stating that their complete film waste was recycled abroad (personal communication with Stotz, 2019-07-10). Rigids from PP could be recycled in CH, however, no respective information was obtained, whereas for the PET rigids and PP films, no recycling infrastructure exists in Switzerland. Thus, it was assumed that 10% of the collected waste of PP and PET rigids and PP films were as well utilized in cement kilns and the rest was recycled abroad.

#### 2.8.1-I. C&I collection systems – hospitality packaging

OUT from use – IN to separate collection

PET drinking bottles from the hospitality sector are collected via the PET drinking bottle collection (listed under consumer packaging collections) based on personal communication[24]. Some stakeholders did not separately collect plastic packaging waste except for PET drinking bottles. One stakeholder could return its waste to its main food supplier, for which a disposal company takes care of the waste. Based on this obtained information, for the target collection items (see Table 1) apart from re-usable boxes, an amount corresponding to 10% of the waste amount was assumed to be collected, analogously to manufacturing packaging (see Section 2.8.1-G). For re-usable boxes, a collection rate of 50% was assumed for broken boxes (the information that they were recycled was obtained via personal communication with Nadler, 2019-07-17).

OUT from separate collection - IN to sorting CH / sorting abroad / energy recovery in cement kiln:

A portion of the collected waste of LDPE films from hospitality is recycled in Switzerland (see sheets Sorting subsegments / mat. flows of a sort&rec plant). 10% of the remaining portion were assumed to be used as substitute fuel in cement kilns (analogously to manufacturing and retail packaging, see Sections 2.8.1-G and 2.8.1-H). The remainder was modelled to be recycled abroad. For the other collected subsegments, no information

[23] This was confirmed by one retailer, which mentioned that they try to convey their wastes to a sensible disposal, however, this is often challenging and economically often not reasonable (personal communication Stotz, 2019-05-15).

[24] SV Group, Candrian Catering, Tibits, Kantonsspital Luzern

regarding in-country recycling was obtained[25]; therefore, 10% were assumed to go to cement kilns and the remaining 90% to be recycled abroad. The assumption of films and rigids from the hospitality sector being recycled abroad was also based on personal communication with Nadler, 2019-07-17.

### *2.8.2 Building and Construction (B&C)*

OUT from use:

The waste amounts for the individual subsegments, except for *other B&C products*, were calculated bottom-up based on the demolished and renovated floor, window and roof area and the length and renewal rate of pipe networks. These data were combined with the amounts of B&C products made of the considered plastics per area or network length. The consumption amounts per area / network length in the past were needed, as B&C products have long lifetimes. Specifically, as fixed lifetimes for the individual products, based on literature, were used, the consumption amount of the year (2017 minus lifetime) was used for the calculation. For *pipes and ducts*, a further distinction between product groups within this subsegment was made. For the detailed calculation methods and sources, see database sheet B&C bottom-up.

For the subsegment *other B&C products*, a fixed average product lifetime of 33 years was assumed based on Geyer et al. [48]. Consequently, the waste amount in 2017 was equated to the use amount in the year 1984. To calculate the respective use amount in 1984, a linear increase of the plastics use amount was assumed, starting from 0 t in the year 1950 to the estimated use amount in 2017 (27’325 t).

OUT from use – IN to energy recovery in WTE plant:

Apart from the separately collected items, the waste from demolition and renovation is collected together with other waste (e.g. painted wood) in a burnable fraction for incineration in WTE plants (personal communication with Van der Haegen, 2019-04-30; [61]; [62]).[26] The amount that was sent to WTE plants was calculated as the difference between “OUT from use” and “IN to separate collection”. Some pipes might remain in the ground after the end of their use phase, which was not modelled due to missing data (the respective amounts would be included in the amounts specified as incinerated in WTE plants).

OUT from use – IN to separate collection:

---

[25] They might be included, though, in the amounts of HDPE rigids recycled in Switzerland specified on sheet mat. flows of a sort&rec plant, which were completely allocated to *C&I Manufacturing*, which was neglected.

[26] The small share of plastics potentially collected in other fractions (e.g. mixed demolition waste or inert fraction, which are partly landfilled) was neglected.

For insulation materials, the total separate collection amount was available from Wiprächtiger et al. [14]. That research work distinguished between bulky-goods collection and single-material collection (for waste from demolition and renovation combined). For the other product subsegments, the different collection pathways are described here below. General information on the single-material collection at renovation was available from GEO Partner et al. [51].

#### 2.8.2-A. single-material collection at selective demolition

<u>OUT from use – IN to separate collection:</u>

From one demolition company, information was obtained that certain parts are removed prior to demolition of a building, for subsequent energy recovery (personal communication with Van der Haegen, 2019-04-30). Such parts include windows, as well as flooring and roof lining if the respective area is big enough (e.g. from industrial buildings), and pipes depending on accessibility and size. Another demolition company also collects plastic items (windows, doors, insulation material) in a single-material collection for re-use purposes (personal communication with Hiltbrunner, 2019-04-30). Based on this information, it was assumed that a very small share (1%) of the waste from the mentioned subsegments was separately collected at demolition.

<u>OUT from separate collection – IN to energy recovery in WTE plant:</u>

*Pipes* and *window profiles* from domestic installation collected in the single-material collection at selective demolition of buildings are incinerated (personal communication with Van der Haegen, 2019-11-29 and 2019-04-30).[27] Windows are partially re-used (personal communication with Hiltbrunner, 2019-04-30); however, this was neglected due to missing data. It was assumed that the other items from the single-material collection at selective demolition were incinerated as well. The incineration was assumed to take place in WTE plants rather than cement kilns, due to the high PVC content of the waste. Chlorine contained in alternative fuels causes operating problems in cement kilns [52] and the chlorine content in certain types of cement is limited [53].

#### 2.8.2-B. bulky-goods collection at demolition / renovation

<u>OUT from use – IN to separate collection:</u>

Demolition and renovation plastic waste was collected in a burnable fraction (see "OUT from use – IN to energy recovery in WTE plant" above), in single-material collection streams (see Sections 2.8.2-A and 2.8.2-C) or in the bulky-goods collection. Based on information obtained from demolition companies (personal communication with Van der

[27] The reason for their separate dismantling is assumed to be energy recovery, as well as the higher purity degree of the other separated fractions that can be gained in this way, resulting in, for example, higher inertness.

Haegen, 2019-04-30; personal communication with Hiltbrunner, 2019-04-30), it was assumed that for both demolition and renovation, half of the waste not collected in a single-material collection was collected in the bulky-goods collection (the rest going to WTE plants). These shares can be found on the sheet B&C bottom-up.

OUT from separate collection – IN to sorting CH:

The complete bulky-goods collection is conveyed to bulky-goods sorting plants in Switzerland (personal communication with Van der Haegen, 2019-04-30).

#### 2.8.2-C. single-material collection at renovation

OUT from use – IN to separate collection:

*Pipes and ducts:*

800–1000 t of underground cable conduits were collected at a recycling plant in Switzerland (personal communication with Tonner, 19-07-16). An average of 900 t was used for the model, with two-thirds modeled as HDPE and one-third as PP (based on personal communication with Tonner, 2019-07-16). Pipes were also separated in bulky-goods sorting for recycling (personal communication with Staubli, 2020-03-05). It was assumed that half of the 900 t stemmed from bulky-goods sorting, and the other half was collected separately. Underground pipes are generally not deconstructed, only renovated [12]. Therefore, the collected waste from underground pipes is listed here under renovation, not under demolition.

Regarding PVC-pipe waste from Switzerland, mainly pre-consumer waste (cut-offs) is recycled according to the authors' knowledge[28], as post-consumer waste is rather incinerated due to contaminations and economic reasons (personal communication with Burkhalter, 2018-12-06 and 2018-12-10). The few collected underground PVC pipes, which were exported for recycling or incinerated (personal communication with Tonner, 2018-12-06), were neglected.[29]

*Flooring:*

A nationwide collection system for PVC flooring exists in Switzerland. The collection is conducted solely by ARP Schweiz (Arbeitsgemeinschaft für das Recycling von PVC Bodenbelägen) (personal communication with Helminiak, 2018-12-07, [54]). The total

[28] Pipes were also collected in the past by the Re-Log system [46], but now almost no plastics are collected by the Re-Log system anymore except for non-consumer films from retail (personal communication with Senn, 2019-05-23). Also, a recycling initiative for PVC pipes existed in CH in the past [74]; however, the respective recycling chain is no longer in place (personal communication with Wiederkehr, 2019-05-31).

[29] In Germany, the Kunststoffrohrverband (KRV) [75] is operating a collection and recycling system for pipes (including pre- and post-consumer gas and drinking water pipes and cable conduits from HDPE, sewage pipes from PP or PVC, and drainage pipes from PVC [76]). At least for PVC pipes, besides cut-offs, also post-consumer pipes are recycled by this system [77].

amount was recycled at one plant located in Troisdorf, Germany, where a combined amount of 2500 t of PVC flooring collected from Germany, Austria, France and Switzerland was managed in 2017 (personal communication with Zimmermann, 2018-12-04). As it was unknown which share of the total collection amounts of the other countries than Switzerland is recycled at the plant in Troisdorf, it was assumed that an equal amount stemmed from each country, resulting in a Swiss collection amount of 625 t.[30]

*Window profiles:*

Roughly 2000 t of post-consumer window profiles were collected in 2017 (personal communication Röschli, 2019-04-29).[31] The collection was carried out by some window selling companies [51] when they install new windows, as well as by disposal companies (personal communication with Steinauer, 2018-12-05). Also in bulky-goods sorting plants, window profiles are separated for recycling (personal communication with Staubli, 2020-03-05). It was assumed that 75% of the total collection amount, i.e. 1500 t, were separated from the bulky-goods collection, and therefore 500 t were separately collected in the single-material collection. This assumption was based on the feedback from the contacted stakeholders separately collecting windows that collect only small amounts (personal communication with Stalder, 2018-12-05, personal communication with Diebold, 2018-12-06, personal communication with Steinauer, 2018-05-12).[32] The collection amount was broken down on the plastic-type basis according to Ecoinvent [55].

*Roof lining:*

20 t of PVC roof lining waste from renovation were delivered by a number of stakeholders to Sika Supply Center Sarnen, which is the only entity collecting PVC roof lining in Switzerland (personal communication with A. Schaub, 2018-12-04).[33] No roof lining from other considered plastics is collected in Switzerland.

<u>OUT from separate collection – IN to sorting abroad:</u>

---

[30] Neither ARP, nor the German related organization AgPR (Arbeitsgemeinschaft PVC-Bodenbelag Recycling) (AgPR (2020)) publish the Swiss collection amount. ARP only stated that the collected amounts have increased drastically since the initiation of the collection system 20 years ago and have been constantly high since 10 years (personal communication with Helminiak, 2018-12-07).

[31] According to personal communication with Röschli, 2019-04-29, roughly 2000–2500 t/a of window profiles were collected. This amount might include a share of pre-consumer waste; therefore, the lower value was used.

[32] Sometimes the windows are also given away for re-use.

[33] In the past, the collection amounts were higher. Ten years ago, about 200–250 t/a were collected. Today, the cost for disposal in WTE plants is much lower (personal communication with A. Schaub, 2018-12-04). The collected amounts today mainly stem from Ticino.

Except for the collected piping, which is sorted in Switzerland, the material collected via a single-material collection at renovation is completely exported for sorting and recycling.[34]

<u>OUT from separate collection – IN to sorting CH:</u>

The pipes from the single-material collection at renovation undergo sorting in Switzerland.

### *2.8.3 Automotive*

<u>OUT from use:</u>

The total amount of considered plastics in EoL vehicles arising in Switzerland was calculated from the amount of considered plastics in exported EoL vehicles (see Section 2.2.3) and the share of exported EoL vehicles of all EoL vehicles calculated from SARS [56]. To calculate the amounts of plastics in the exported EoL vehicles, a lower total plastic share than that of vehicles produced in 2017 was used due to the increasing plastic share over time (11% instead of 15%; see sheets Intl. trade parts and products and variable parameters). To allocate the total plastic waste amount to the individual plastic types, the same shares as for vehicles produced in 2017 from sheet Plastic types were used.

<u>OUT from use – IN to energy recovery:</u>

No EoL vehicles from Switzerland are incinerated without prior treatment.

<u>OUT from use – IN to separate collection CH:</u>

All EoL vehicles disposed of in Switzerland must be delivered to an authorized dismantling facility [57]. The share of EoL vehicles disposed of in Switzerland was calculated from SARS [56]. The total amount was allocated to the individual plastic types using the same shares as for vehicles produced in 2017 from sheet Plastic types.

<u>OUT from use – Export EoL vehicles:</u>

For the calculation procedure of the amounts of plastics in exported EoL vehicles, see Section 2.2.

<u>OUT from use – Unknown destiny EoL vehicles:</u>

---

[34] For *Flooring*, the collection system is financed by the flooring producers abroad (personal communication with Helminiak, 2019-10-25), the related recycling system is operated by AgPR. The *Window profiles* collection is connected to the Rewindo system and conveyed to the VEKA Umwelttechnik recycling plant in Germany (personal communication with Steinauer, 2018-12-05; personal communication with Röschli / Vetter, 2019-04-18). The Swiss roof lining collection is also export to Germany (company FG Kunststoffmatten) (personal communication with Arz, 2018-12-04), the respective international organization is Roofcollect. For further information, see “IN to separate collection” above in this section.

EoL vehicles with unknown destiny might correspond to illegal exports; however, no related data is available. The amounts of EoL vehicles with unknown destiny were calculated based on SARS [56] as the difference between the total EoL vehicles (see “OUT from use”) and the sum of EoL vehicles collected in Switzerland (see “OUT from use – IN to separate collection CH”) or exported (see “OUT from use – Export EoL vehicles”).

<u>OUT from separate collection – IN to sorting CH:</u>

All EoL vehicles collected in Switzerland undergo sorting in Switzerland (see “OUT from use – IN to separate collection CH”).

#### 2.8.4 Electrical and Electronic Equipment (EEE)

<u>OUT from use:</u>

The EEE waste amounts were calculated as the sum of WEEE undergoing energy recovery, separately collected WEEE and exported WEEE.

<u>OUT from use – IN to energy recovery in WTE plant:</u>

A small share of WEEE is disposed of in the residual waste collection (going to WTE plants). This amount was calculated from the share of WEEE in the total amount of municipal solid waste from BAFU [58].[35] The total amount was distributed to the subsegments, assuming that the same share of each subsegment is mis-collected in the residual waste instead of being separately collected.

<u>OUT from use – IN to separate collection:</u>

The separate collection amounts were calculated based on data on the total collection amounts of the individual subsegments from a database regarding WEEE disposal in Switzerland [59]. Additional data on the shares of total plastics and individual plastic types in the individual subsegments (as specified on sheet variable parameters) were used.

<u>OUT from use – Export WEEE:</u>

The export amounts of WEEE were calculated on sheet Intl. trade parts and products (see Section 2.2). Only exported WEEE products were regarded here, not WEEE parts (the latter were assumed to be exported from sorting – see Section 2.10.4).

<u>OUT from separate collection – IN to sorting CH:</u>

The complete separate WEEE collection is sorted in Switzerland [60].

---

[35] Batteries (1’000 t) were neglected due to the small share of plastics contained within.

### 2.8.5 Agriculture

OUT from Use:

The waste amounts were calculated based on the lifetime of different agricultural products known from Kalberer et al. [6] and the use amounts of the respective products in the past years. The latter were estimated based on the use amounts in 2017, using the temporal development of the total Swiss plastic consumption as a proxy. Calculation details can be found on the sheet agriculture data.

OUT from use – IN to energy recovery in WTE plant:

The amounts going to energy recovery were calculated as the difference between OUT from Use and IN to separate collection. It was assumed that the energy recovery took place in WTE plants, since no preparation as alternative fuels for cement kilns was known to occur.

OUT from use – IN to separate collection CH:

About 1500 t of silo wrapping films were collected by roughly 70 stakeholders[36] in Switzerland (personal communication with Tonner, 2018-11-08 and 2019-07-16). Additionally, about 100 t of greenhouse films were collected (personal communication with Tonner, 2019-04-09). Besides these, no other agricultural plastics waste is separately collected according to the knowledge of the authors. One collection stakeholder stated that WTE plant disposal was cheaper than recycling, and as such the separately collected amount was decreasing yearly (personal communication with Senn, 2019-05-23).

OUT from separate collection – IN to sorting CH / sorting abroad:

The separately collected materials are all transported to the same recycling plant in Switzerland (personal communication with Tonner, 2018-11-08; personal communication with Senn, 2019-05-23), and then partly exported for recycling (personal communication with Tonner, 2018-11-08). The exported amounts fluctuate based on price and demand conditions (personal communication with Tonner, 2018-11-08). It was assumed that half of the collection was sorted in Switzerland, half abroad.

### 2.8.6 Household items, furniture, leisure and others

OUT from use:

The waste amount and composition were modelled to correspond to the use amount and composition, assuming a fixed lifetime of 5 years based on Geyer et al. [48] and no considerable change in the use amount and composition over the last 5 years. The latter is

[36] including RESI [45], Senn Brunnen (personal communication Senn, 2019-05-23), Ziegelgut [78]

based on the fact that the development of the total net import of plastics in primary forms and plastic products (listed under the tariff heading 39 – “Plastics and articles thereof”) was rather constant over the last 5 years [2].

OUT from use – IN to energy recovery:

Energy recovery for this product segment includes hazardous waste incineration (e.g. of medical waste). It was assumed that no mixed waste was used as substitute fuel in cement kilns. The path through which the plastics reach the energy recovery process is mentioned on sheet Collection subsegments.

OUT from use – IN to separate collection CH:

Information on relevant collection systems and collected amounts with related assumptions is given directly on sheet Collection subsegments.

OUT from separate collection CH – IN to sorting CH / abroad:

The *household items* and *toys* collected via mixed-plastic collection bags are sorted abroad (along with the rest of the respective collection, see Section 2.8.1-C for further related information). The items collected in the bulky-goods collection are sorted in Switzerland (along with the bulky goods collection of B&C products, see Section 2.8.2).

### *2.8.7 Textiles*

OUT from use:

The average lifetime of textiles is 5 years according to Geyer et al. [48]. It was assumed that there was no significant change in quantities and polymer types in the last 5 years, for the same reason as described in Section 2.8.6. Therefore, the total waste amount was assumed equal to the total use amount for each textile subsegment, except for *agrotextiles*. This research work neglects what may be a longer lifetime for *textile flooring*, *textile furniture* and *mobility textiles*. For *agrotextiles*, a more detailed modelling was done, as for the other agriculture subsegments (Section 2.8.5).

OUT from use – IN to energy recovery in WTE plant:

All textiles that are not separately collected, exported or have unknown destiny are assumed to be incinerated in WTE plants. Details of how each textile subsegment is conveyed to energy recovery are specified on sheet Collection subsegments.

OUT from use – Export / Unknown destiny along with EoL vehicles:

A share of the *mobility textiles* is exported or has an unknown destiny, along with the EoL vehicles that they are contained in. The respective amounts were calculated as the differences between the waste amounts and the collected amounts (no vehicles were sent directly from use to energy recovery). These amounts were then split between the two

pathways based on the ratio of the amounts of plastics in the automotive segment that were exported and that had an unknown destiny.

OUT from use – IN to separate collection CH:

The separate collection systems for textiles and the main considerations regarding the collected amounts are described here below. No *agrotextiles* and *other technical textiles* are collected according to the authors' knowledge. Further details on the textiles collection are reported in the database sheet Collection subsegments.

#### 2.8.7-A. apparel and household textiles collection

OUT from use – IN to separate collection CH:

*Apparel* and *household textiles* are collected together via the respective collection schemes operated by TEXAID [61], Tell-Tex [62], and Caritas [63]. The separately collected share of the total *apparel* waste amounted to 34% [3], the same share was assumed for the collection of *household textiles*.

OUT from separate collection CH – IN to sorting CH:

For all schemes, a part of the collection is sorted in Switzerland [64]–[66]. As data on the exact amounts sorted in Switzerland and abroad were scarce, and one collection scheme has sorting facilities in various other countries besides Switzerland [65], a share of 19 % was assumed to be sorted in Switzerland based on Haupt et al. [41].

OUT from separate collection CH – IN to sorting abroad:

Based on the collection share sorted in Switzerland (19%, [77]), 81 % of the collection are modelled to be sorted abroad.

#### 2.8.7-B. bulky-goods collection

OUT from use – IN to separate collection CH:

Half of all *textile flooring* and *textile furniture* waste was assumed to be collected via the bulky-goods collection, along with wastes from the B&C and Household items, furniture, leisure and others segments. This relatively high share was chosen as many items belonging to the two concerned textile subsegments are large in size. The collection can take place via recycling centers or at selective demolition.

OUT from separate collection CH – IN to sorting CH:

The bulky-goods collection is completely sorted in Switzerland (see Section 2.8.2-B).

#### 2.8.7-C. EoL vehicle collection

OUT from use – IN to separate collection CH:

The pathways for the waste of *mobility textiles* are the same as for the EoL vehicles in which they are contained: the *mobility textiles* that are not contained in exported EoL vehicles or vehicles with unknown destiny are entirely separately collected.

OUT from separate collection CH – IN to sorting CH:

All EoL vehicles collected in Switzerland including the contained *mobility textiles* undergo sorting in Switzerland (see 2.8.3).

## 2.9. Sorting segments

The total sorting amounts for each segment are compiled on this sheet from the sheet Sorting subsegments. For further information, see Section 2.10.

## 2.10. Sorting subsegments

In this section, the sorting processes and pathways of the exiting material flows are described. For the amounts from the different collections entering the individual sorting processes (“IN to sorting CH / abroad”), see “OUT from separate collection” in Section 2.8. The amounts entering the sorting processes are also reported in the sheet Sorting subsegments.

### *2.10.1 Packaging*

The sorting losses of the individual plastic types in the individual product subsegments and the respective data sources for the sorting processes of all packaging collections (except for the part of the hollow bodies collection that was sorted in Switzerland) are provided in the sheet Recycling subsegments.

The part of the hollow bodies collection sorted in Switzerland is either sorted using NIR technology followed by a float-sink sorting (involving shredding and washing) (Case 1), or by a float-sink sorting directly (Case 2) (see Section 2.8.1-B). For the float-sink sorting (both cases), the sorting losses of the target polyolefin plastics (i.e. the PE and PP items contained in the collection) were known from a Swiss recycling plant (as specified on sheet Sorting subsegments). The polyolefins retrieved from float-sink sorting in Switzerland, for both cases, were recycled in Switzerland. Non-polyolefin plastics, i.e. the PET and PS items contained in the collection, are sorted out and used for energy recovery. This is relevant for Case 2, as in Case 1 these plastic types are separated by the precedent NIR sorting.

For the share of the hollow bodies collection which is additionally sorted via NIR sorting (Case 1), the additional NIR sorting losses of the polyolefins were estimated based on the total losses in the entire float-sink sorting-recycling-chain, as no relevant data were

obtained from the NIR sorting plants. For the polyolefins, the total losses in Case 1 (including precedent NIR sorting) were assumed to be higher than in Case 2, because the NIR sorting is applied to the complete products so that mis-sorting can occur, e.g. due to labels/sleeves or the target plastics being present as composites (even if mechanically separable). In contrast, the float-sink sorting is applied to shredded and washed materials, therefore, sorting losses are assumed to arise only where target materials adhere to foreign substances or are present as mechanically non-separable composites.[37] The calculation procedure for obtaining the value for the NIR sorting losses took into account these considerations, as outlined in the sheet Sorting subsegments. The same NIR sorting loss rate as for polyolefins was assumed for the PET and the PS contained in the hollow bodies collection. The calculation resulted in lower overall losses in Case 1 (involving NIR sorting), compared to Case 2 (mere float-sink sorting), since also PET and PS can be recovered in Case 1.

The PS and PET fractions resulting from NIR sorting were assumed to be recycled abroad (except for PET *food bottles*). This assumption was taken, because among PET waste, only *food bottles* are recycled in Switzerland, and among post-consumer PS waste, only EPS recycling takes place in Switzerland, according to the knowledge of the authors. PET *food bottles* from the hollow bodies collection were assumed to be recycled in Switzerland, although the gained secondary materials were not used for the production of *food bottles* (personal communication with Müller, 2019-07-04). The HDPE and PP amounts sent for recycling in Switzerland after the NIR sorting were based on industry data (1494 t out of 2322 t total input into NIR sorting, after losses) (personal communication with Tonner, 2019-04-09), and with these, the amounts of HDPE and PP exported for recycling could be calculated. For the plastic fractions from NIR sorting recycled in Switzerland, sorting losses also arise in the subsequent float-sink sorting taking place at the recycling plant (see second paragraph of this section). These sorting losses were subtracted from the NIR sorted amount to calculate the mass entering the recycling process. For the NIR sorted material recycled abroad, additional sorting losses arising at the recycling plant are included in the recycling losses (see sheet General information regarding the allocation of the sorting / recycling losses).

The sorting losses are conveyed to energy recovery either in WTE plants or in cement kilns, the respective shares and sources are given on sheets Sorting subsegments and Recycling subsegments.

---

[37] In float-sink sorting, it can, however, occur that a mechanically non-separable composite partly consisting of non-target plastic types has an overall density that is as low as to cause the composite to float, leading to non-target plastic types ending up in the target float fraction. This is reflected by the limited products that can be made from the resulting secondary materials.

### 2.10.2 Building and Construction (B&C)

OUT from sorting CH – IN to recycling CH:

The separately collected pipes that are sorted in Switzerland are also recycled in Switzerland. Losses in the applied sink-float process were known from personal communication with Tonner, 2018-12-06 (see sheet Sorting subsegments). In addition, a share of the pipes collected via the bulky-goods collection is separated for recycling at the bulky goods sorting taking place in Switzerland. It was assumed that polyolefin pipes separated in the course of the bulky goods sorting were completely delivered to the recycling plant in Switzerland where also the directly separately collected pipes were recycled, and that no PVC pipes were separated. For the pipes conveyed to recycling in Switzerland from bulky goods sorting, subsequent float-sink sorting losses arise as for the separately collected pipes. The total amount entering the respective float-sink sorting process (from both separate collection and bulky goods sorting), as well as the respective sorting losses, were known (see description to *pipes and ducts* under Section 2.8.2-C).

OUT from sorting CH – IN to recycling abroad:

Besides pipes, also window frames are separated for recycling in bulky-goods sorting. These are conveyed to a recycling plant abroad, since no post-consumer window profiles are recycled in Switzerland. The respective amount was estimated to be 1500 t (see Section 2.7), and it was assumed that no subsequent sorting losses arise (as described for the other B&C specific separate collections that were sorted abroad, see “OUT from sorting abroad – IN to recycling abroad”).

OUT from sorting CH – IN to energy recovery:

The complete plastics from bulky-goods sorting, except for the separated pipes and window profiles, are sent to energy recovery in a WTE plant. The losses from the pipes sorting (see “IN to recycling CH”) are sent to energy recovery in WTE plants and cement kilns (the shares were known from personal communication with Tonner, 2019-04-09).

No post-consumer insulation materials made from plastics are recycled in Switzerland [14]. There is no separate collection for recycling, nor are insulation materials sorted out for subsequent recycling in bulky-goods sorting. One barrier is the presence of the brominated flame retardant hexabromocyclododecan (HBCD) in insulation materials produced before 2015 [14], which would require the application of a specific process allowing for the removal of this hazardous substance (like the CreaSolv® process [67]). Besides, glue and mineral residues hamper recycling [14].

OUT from sorting abroad – IN to recycling abroad:

It was assumed that no additional sorting step is required prior to the sorting procedures taking place at the recycling plants, since the respective collections are already product- and material-specific. This means that no sorting losses occur at this point.

### 2.10.3 Automotive

The end-of-life vehicles (ELVs) delivered to the licensed dismantlers in Switzerland first undergo decontamination [56], [57]. Then, valuable, large and easily accessible parts are dismantled for re-use or recycling; the remaining parts of the vehicles are shredded [56], [57].

OUT from sorting CH – IN to recycling abroad:

Among the plastic parts in vehicles, bumpers are the only part being dismantled for recycling today in Switzerland; however, this is not a common practice (personal communication with Leimgruber, 2019-10-03; personal communication with Kaufmann, 2019-09-13; personal communication with Tonner, 2018-12-06). The respective recycling takes place abroad, such as at the German company WIPAG (personal communication with Tonner, 2018-12-06). The amount of bumpers dismantled for recycling was calculated assuming that they only consist of PP (blended with ethylene propylene diene monomer (EPDM) rubber, which is not considered here) [68], [69]. It was assumed that 90% of all PP exported in EoL bumpers entered recycling, while 10% were exported for re-use. These shares seem plausible, as therewith the shares of parts recycling and total parts re-use roughly match the recycling and re-use shares of all dismantled automotive parts in Germany [70]. The export numbers were determined as described in Section 2.2.3; however, it is possible that EoL bumpers meant for recycling are not exported under the tariff heading for bumpers (8708.10), but under the tariff heading for plastic waste (3915).[38]

OUT from sorting CH – Export as second-hand parts:

In addition to the bumpers dismantled for recycling, plastic parts are dismantled for re-use abroad or in Switzerland. This includes plastics in vehicle electronics (which are in-

---

[38] According to the government directives, also other vehicle parts that made from plastics, more specifically fuel tanks and mud/splash guards, shall be dismantled in Switzerland [79]. However, from the most advanced vehicle dismantling plant in Switzerland (according to their own description), information was obtained that they are the only one in Switzerland with the infrastructure needed for dismantling the mentioned parts and that these parts are not actually dismantled due to missing economic profitability (personal communication with Leimgruber, 2019-10-03). Also, a large Swiss organization providing automotive spare parts stemming from dismantling mentioned that the dismantling of plastic parts is not economically viable (personal communication with Kaufmann, 2019-09-13). Both stakeholders mentioned that a recycling fee could create financial feasibility. Kaufmann also mentioned that the variety of plastics used in automotive is large and that the dismantling is sometimes laborious (e.g., plastic bumpers can be fixed with many screws, whereas metal bumpers are only suspended via four mounts). Further automotive plastic parts that are or have been partly dismantled in other countries for recycling include radiator grilles and hubcaps [68]; but for the same reasons, this has only been done to a limited extent [68], [70]. Other parts with potential for dismantling are ram protection strips, engine covers, other plastic parts of the engine compartment like air intake modules or air filter housings, and wheel arch liners [68]. Additional parts which might be suitable for dismantling and recycling include front and rear fenders, lead-acid battery cases, interior dashboards or car door panels [80].

cluded in the automotive segment, see sheet Product (sub)segments). The amounts of exported automotive EoL parts made from the different plastic types were taken from sheet Intl. trade parts and products (see Section 2.2.3). For PP, the amounts assumed to be exported as bumpers for recycling (see above) were subtracted from the total PP export amount. Thereby, the exported automotive EoL parts were considered as from dismantling facilities only. Retrieval from repair or service replacements was not assumed, because parts exchanged in this context are usually broken or not functional anymore. No exported WEEE parts (also listed on sheet Intl. trade parts and products) were allocated to automotive EoL parts, although exported WEEE parts declared under the EEE-related tariff headings may be from vehicles. Regardless, the total amount of exported WEEE parts is relatively small.

<u>OUT from sorting CH – IN to use in CH:</u>

Besides the exported parts, dismantled parts are assumed to also be used in Switzerland as spare parts. Since no related information was available, it was assumed that the same amounts of ELV parts as exported for re-use were re-used in Switzerland. This choice was based on the considerations that the requirements for spare parts might be higher in Switzerland than in other countries, but on the other hand, re-use within the country is easier because there is no need for transportation and export formalities.

<u>OUT from sorting CH – IN to energy recovery in WTE plant:</u>

The plastics remaining in the ELVs after dismantling, enter the shredding process and end up to a large extend in the shredder light fraction (RESH), which in Switzerland is sent to WTE plants [56].[39] The respective amount was calculated as the difference of the total plastics in ELVs and the dismantled parts. Plastics ending up in other fractions were neglected; however, also their energy content might be used in practice, if they are subsequently sorted out for incineration or, e.g., end up in metal smelters.

#### *2.10.4 Electrical and Electronic Equipment (EEE)*

After decontamination (removal of parts containing hazardous substances), WEEE sorting consists of (partly repeated) dismantling and shredding steps. After sorting, the plastics contained in WEEE end up in dismantled parts for re-use in Switzerland or abroad, in a plastic fraction, in a plastic-metal fraction, or as contaminations in other fractions.

<u>OUT from sorting CH – IN to recycling abroad:</u>

---

[39] ELVs are partly shredded together with WEEE [80]–[84]. Generally, there are plants for the recycling of plastics from RESH from ELVs and WEEE, e.g. in the United Kingdom and France [81], [82], [85]–[87]. HDPE, PP, PS and ABS are recovered in such processes [81]. However, automotive shredder residue is classified as a hazardous waste, as it routinely contains, e.g., heavy metals [82], [88]. The resulting plastic products can contain extraneous matter, and stem from various car makers, models, and vintages [82]. They can only be used for undemanding applications [82].

The plastic fraction is completely exported for recycling according to the information available (personal communication with Gasser, 2019-01-22). Recycling takes place, among others, at MGG Polymers in Austria [71]. The total amount of the plastic fraction was obtained from the Toocy database [59]. For the plastic-metal fraction, the share undergoing further separation was available from the Toocy database [59]. However, this separation might only target the recycling of the contained metals, since in certain European plants, the plastics contained in such plastic-metal fractions from WEEE are at least partly used for energy recovery purposes (personal communication with Dewulf, 2019-11-07). Based on personal communication with Hummen, 2020-02-13, a share of 25% of the plastics in the plastic-metal fraction undergoing further separation was assumed to be subsequently recycled. The share of plastics in the total plastic-metal fraction was, due to lack of data, assumed to amount to 50 %, which roughly matched the data from a batch test of boilers (personal communication with Hummen, 2020-02-13). However, this share is product-specific, as the separation efficiency of plastics and metals – determining which amounts end up in the mixed plastic-metal fraction – depends on the specific combination of plastic types and different metals.

Starting from the total amount of plastics conveyed to recycling, the respective amounts for the individual subsegments were calculated. For the *cooling, refrigerating and AC devices* subsegment, the amount of the plastic fraction was obtained from SARS [10], and for the *ICT equipment and CE* subsegment, the share of the plastic-metal fraction was obtained from Swico et al. [60]. From that information, the amount of total plastics from the collection conveyed to recycling was calculated for the two subsegments (see sheet Sorting subsegments for the calculation). For the subsegment *ICT equipment and CE,* only the total amount of the plastic-metal fraction was available; it was assumed that the same share of this fraction as for the overall plastic-metal fraction is further separated, and that of the amount undergoing further separation, 25% of the contained plastics are recycled (analogously as described above for the overall plastic-metal fraction).

The rest of the total plastics amount conveyed to recycling was attributed to the other three subsegments in a way that the same share of plastics in the collection goes to recycling for each of those subsegments. The plastic-type shares in the total amount of plastics conveyed to recycling for each subsegment, in the model are the same as the shares in the collection (Section 2.8.4), since no plastic-type-specific sorting takes place at this stage (beside the small share of dismantled parts, which was neglected).

<u>OUT from sorting CH – Export dismantled WEEE parts for re-use:</u>

The exported WEEE parts (see sheet Intl. trade parts and products) were assumed to stem from WEEE dismantling. No allocation to subsegments was done (see Section 2.2 of the *Data in Brief* article), even though probably, based on the assembly of the electrical and electronic products, different shares of parts in specific subsegments are dismantled. This was neglected, since the total amount of such parts is very small.

OUT from sorting CH – IN to use in CH:

It was assumed that the same amounts of dismantled parts from WEEE were re-used in Switzerland as were exported for re-use. On the one hand, aesthetical and technical requirements in Switzerland may be higher than in other countries, but on the other hand, a re-use in Switzerland is also less complicated than an international sales process.

OUT from sorting CH – IN to energy recovery:

The plastics in the part of the plastic-metal fraction not undergoing further separation, and a share of the plastics in the further separated plastic-metal fraction, were assumed to be incinerated (see "IN to recycling abroad" above for further details). Additionally, if plastics are not dismantled as WEEE parts, nor end up in the plastic or the plastic-metal fraction, they end up in other fractions and are assumed to be incinerated, e.g., in metal smelters. It was assumed that in all mentioned cases, the energy content in the plastics is recovered and the respective amounts were listed correspondingly. The amounts of the plastic types used for energy recovery, for each subsegment, were calculated as the differences between the amounts entering sorting and the amounts entering recycling or re-use. Thereby, for the sake of convenience, the complete re-use amount of each plastic type (comparatively very small) was subtracted from the *other EEE* subsegment. Note that even if the sorting takes place in Switzerland, energy recovery from the plastics can also take place abroad when the respective product fractions are treated abroad.

### *2.10.5 Agriculture*

OUT from sorting CH / abroad – IN to recycling CH / abroad / energy recovery in WTE plant / cement kiln:

The losses in the sorting processes in Switzerland and abroad are provided in the sheet Recycling subsegments. The same sheet contains the shares for which energy recovery takes place in WTE plants or cement kilns and the related data sources. Sorting in Switzerland takes place in the same plant as for, e.g., non-consumer packaging films. For this plant, the overall losses were available (specified on sheet Sorting subsegments). For sorting abroad, the same losses were assumed for agricultural films as for non-consumer packaging films.

### *2.10.6 Household items, furniture, leisure and others*

OUT from sorting CH – IN to energy recovery in WTE plant:

*Furniture* and *sports items* from the bulky-goods collection are sorted in the bulky-goods sorting plants that also process B&C bulky-goods waste, and completely conveyed to a

product fraction destined for energy recovery in WTE plants (personal communication with Staubli, 2020-03-05). The variety of plastics arriving at the bulky-goods sorting plants is extremely large, and only plastics from certain B&C applications are separated for material recovery (see Section 2.10.2) (personal communication with Staubli, 2020-03-05).

OUT from sorting abroad – IN to recycling abroad / energy recovery in WTE plant / cement kiln:

The sorting losses for *household items* and *toys* collected in mixed-plastic collection bags sorted abroad and respective data sources, as well as the amounts conveyed to thermal treatments (WTE plants / cement kilns) and respective data source, are provided in the sheet Recycling subsegments.

#### *2.10.7 Textiles*

The flow paths from textiles sorting, with the respective amounts and data sources, are provided in the sheet Sorting subsegments. No textiles from Switzerland are conveyed to mechanical recycling (including a melting process, see definition used in this research work given on sheet General information of the database) according to the authors' knowledge. Instead, *apparel* and *household textiles* are re-used (mainly abroad, but also in Switzerland), or converted to other products like cleaning rags [61], or possibly also insulation materials [72] or padding [73] (the latter is included in the amounts labeled as re-use in the database).

### 2.11. Recycling segments

On the sheet Recycling segments, it is shown which amounts of secondary materials are available for the individual plastic types in the individual segments, and in which segments they are used. The respective amounts were calculated bottom-up, as described under Section 2.12.

The recycling rates of the individual segments – defined as the total secondary materials from a segment divided by the total waste from the same segment – are also provided on this sheet. EEE has the highest recycling rate. An expert with in-depth knowledge of the EEE recycling system in Switzerland, involved in monitoring activities, considered the respective value matching the reality (personal communication with Gasser, 2020-07-08).

### 2.12. Recycling subsegments

The sheet Recycling subsegments in the database provides the amounts of secondary materials from different collection–sorting–recycling pathways, for each plastic type in each product subsegment. In which product subsegments the secondary materials are used in product manufacturing, is also provided in the same sheet. In the following it is described,

how the secondary material amounts were calculated and allocated to the uptaking products. Also the principles, based on which the allocated amounts to product subsegments and manufacturing in Switzerland and abroad were calculated, are outlined.

The amounts of secondary materials were calculated by subtracting the recycling losses from the amounts entering the recycling process. Individual recycling losses were applied for each plastic type in each collection–sorting–recycling pathway. The numbers of the recycling losses, the related data source, and information on the specific recycling process and its location are provided in the sheet Recycling subsegments.[40]

Each secondary material flow of a specific plastic type in a specific product subsegment from a specific collection–sorting–recycling pathway was allocated to the uptaking subsegments. This allocation was done based on a number of data sources on the use of different secondary materials, as well as some assumptions. The data sources and assumptions are specified directly in the sheet Recycling subsegments, next to the concerned values. Information was generally available regarding the subsegments in which the secondary materials from specific collection–sorting–recycling pathways are used, while polymer-specific information was only partially available. When information was only available on a subsegment level, for the individual plastic types, the suitable subsegments – out of all subsegments in which secondary material from a recycling pathway was known to be applied – were assumed based on available information on the use of individual plastic types in individual subsegments. Additionally, certain assumptions were made for the secondary material distribution. For the rigid packaging items undergoing mixed-plastic sorting, it was assumed that the suitable applications for the secondary materials only depend on the plastic type, not the original product. This is considered realistic, since mixed-plastic sorting plants only separate fractions destined for recycling based on plastic type, and do not further distinguish them based on product types. The mixed-plastic collection bags and points were assumed to have a similar composition (see Section 2.8.1-C), and thus, the secondary materials stemming from both collections were assumed to be suitable for the same applications and the related amounts were not separately specified. There are some regulatory restrictions on the use of secondary materials in certain applications. The legal frameworks related to specific product subsegments are mentioned in the sheet Product (sub)segments. Respective restrictions are reflected in the product subsegments taking up the secondary material flows in the model, which are based on data on the current uptake of secondary materials.

---

[40] The sheet Recycling subsegments also contains the sorting losses and related data sources for the packaging (except for the hollow bodies collection sorted in Switzerland) and agriculture segments, as mentioned under Sections 2.10.1 and 2.10.5, since for the packaging segment the sorting losses are plastic-type, subsegment and collection specific.

If a secondary material is used in different applications, the shares used in the individual applications were often unknown. Besides, this distribution may change based on demand conditions. Therefore, in case of missing data, an equal uptake by all suitable subsegments was assumed. In some cases, data were available on the shares of secondary materials used in product manufacturing in Switzerland and abroad. If no such data were available, half of the secondary materials were assumed to be used for product manufacturing in Switzerland, and half abroad for manufacturing of products imported to Switzerland. In this way, secondary materials from Swiss waste recycled abroad are modelled to be imported to Switzerland, directly or in the form of products. In reality, the imported secondary materials stem not only from Swiss waste, but also from waste from others countries. At the same time, the secondary materials from Swiss waste are applied abroad in manufacturing of products used in other countries than Switzerland. However, this discrepancy in the model from reality is acceptable for the purpose of this work, as the applied calculation aims to take into account how much virgin materials can in total be replaced by secondary materials from Swiss waste. The exact origin and fate of the secondary materials become relevant, however, in more detailed studies, e.g. when modeling the flows of hazardous chemical additives in plastics.

The amounts of secondary materials used in product manufacturing were subtracted from the total amount of materials used in product manufacturing to get the needed amounts of virgin materials (see Section 1.3 of the *Data in Brief* article). In very few cases, this resulted in a higher uptake of secondary materials made from a specific plastic type in a specific subsegment than the total amount used in product manufacturing. In such cases, the distribution of the secondary materials was changed so that the entire secondary materials could be taken up by products manufactured in Switzerland or manufactured abroad for Swiss consumption. The adaption of the distribution involved on one hand a different distribution between Switzerland and abroad (only in the cases where no data were available regarding this distribution). In such cases, a note is provided in the database sheet. This concerned

- PET from packaging used in *other products,* and
- ABS from EEE used in *Household items, furniture, leisure and others*.

On the other hand, the distribution among the subsegments taking up the secondary materials was changed, for

- HIPS from the EEE segment, and
- all HDPE secondary material flows allocated to EEE.[41]

---

[41] For the secondary material flow of HIPS, the distribution was adapted so that only 10% of the amount allocated to automotive in the case of an equal distribution (normally, the same share was allocated to each suitable uptaking

product subsegment) was directed to automotive after adaption, and only half of the amount resulting from the equal distribution was allocated to household items, furniture, leisure and other. The rest (90% of the automotive and half of the household items, furniture, leisure and other share from the equal distribution) were allocated to EEE (in addition to the EEE share from the equal distribution). In the same manner, for all HDPE flows suitable for EEE manufacturing, only 10% of the amount resulting from the equal distribution among all suitable subsegments were allocated to EEE and the remaining amount to *pipes and ducts*.

## 3. Literature references


[1] Federal Customs Administration (Eidgenössische Zollverwaltung), “Explanatory Notes to the Customs Tariff - Tares. Chapter 39. Plastics and articles thereof. (Erläuterungen zum Zolltarif - Tares. Kapitel 39. Kunststoffe und Waren daraus.).” 2020.

[2] Swiss Federal Customs Administration (FCA), “‘Swiss-Impex’ database,” 2019. [Online]. Available: https://www.gate.ezv.admin.ch/swissimpex/. [Accessed: 08-Mar-2019].

[3] D. Kawecki, P. Scheeder, and B. Nowack, “Probabilistic Material Flow Analysis of Seven Commodity Plastics in Europe,” *Environ. Sci. Technol.*, 2018.

[4] K. Röschli, “Swiss Plastics Wirtschaftsdaten 2017,” Aarau, 2018.

[5] Swissmem, “Faktenblatt,” 2019.

[6] A. Kalberer, D. Kawecki-Wenger, and T. Bucheli, *Plastik in der Landwirtschaft. Stand des Wissens und Handlungsempfehlungen für die landwirtschaftliche Forschung, Praxis, Industrie und Behörden*, no. 89. 2019.

[7] PlasticsEurope, “Plastics – the Facts 2018,” 2018.

[8] R. Schelker and P. Geisselhardt, “Projekt „Kunststoff-Verwertung Schweiz“. Bericht Module 1 und 2. Studie im Auftrag des Bundesamt für Umwelt (BAFU),” 2011.

[9] WRAP, “PlasticFlow 2025 Plastic Packaging Flow Data Report,” 2018.

[10] S. Ebnesajjad (ed.), *Plastic Films in Food Packaging. Materials, Technology and Applications.* Norwich: William Andrew, 2012.

[11] N. Heeren and S. Hellweg, “Tracking Construction Material over Space and Construction Material Flows,” *J. Ind. Ecol.*, 2018. DOI: 10.1111/jiec.12739.

[12] S. Rubli, Energie- und Ressourcen-Management GmbH, and BAFU, “Bauabfälle in der Schweiz - Tiefbau. Aktualisierung 2015,” 2016.

[13] Lichtensteiger, *Bauwerke als Ressourcennutzer und Ressourcenspender : in der langfristigen Entwicklung urbaner Systeme : ein Beitrag zur Exploration urbaner Lagerstätten*. 2006.

[14] M. Wiprächtiger, M. Haupt, N. Heeren, E. Waser, and S. Hellweg, “A framework for sustainable and circular system design: Development and application on thermal insulation materials,” *Resour. Conserv. Recycl.*, vol. 154, no. August 2019, p. 104631, 2020.

[15] APME, “Plastics. A Material of Choice in Building and Construction. Plastics consumption and recovery in Western Europe 1995,” *Buildings*, 1995.

[16] P. Wäger, M. Schluep, and E. Müller, “RoHS Substances in Mixed Plastics from Waste Electrical and Electronic Equipment. Final Report.,” 2010.

[17] G. Martinho, A. Pires, L. Saraiva, and R. Ribeiro, “Composition of plastics from

waste electrical and electronic equipment (WEEE) by direct sampling," *Waste Manag.*, vol. 32, no. 6, pp. 1213–1217, 2012.

[18] E. Dimitrakakis, A. Janz, B. Bilitewski, and E. Gidarakos, "Small WEEE : Determining recyclables and hazardous substances in plastics," *J. Hazard. Mater.*, vol. 161, pp. 913–919, 2009.

[19] SENS, Swico, and SLRS, "Fachbericht 2018," 2018.

[20] Swiss Federal Council, *Ordinance on the Avoidance and the Disposal of Waste (Waste Ordinance, ADWO). Art. 3 (a)*. Bern, Switzerland, 2019.

[21] Verein PET-Recycling Schweiz, "Stoffstrom von PET-Recycling Schweiz. Stoffstrom 2018.," 2020. [Online]. Available: https://www.petrecycling.ch/de/wissen/zahlen-fakten/stoffstrom. [Accessed: 18-Feb-2020].

[22] Verein PET-Recycling Schweiz, "Was sammeln?," 2021. [Online]. Available: https://www.petrecycling.ch/de/sammeln/was-sammeln. [Accessed: 09-Apr-2021].

[23] F. Dinkel, T. Kägi, R. Bunge, T. Pohl, and A. Stäubli, "KuRVe (Kunststoff Recycling und Verwertung). Ökonomisch-ökologische Analyse von Sammel- und Verwertungssystemen von Kunststoffen aus Haushalten in der Schweiz. Kurzbericht.," Basel: Carbotech, UMTEC, 2017.

[24] Migros, "Infografik Plastik-Flaschen Recycling.," 2020. [Online]. Available: https://generation-m.migros.ch/de/nachhaltig-leben/infografiken/plastik-flaschen-recycling.html. [Accessed: 19-Feb-2020].

[25] Coop, "Richtiges Recycling leicht gemacht. Diese Wertstoffe nehmen wir bei uns zurück.," 2020. [Online]. Available: https://www.taten-statt-worte.ch/content/dam/act/TatenstattWorte_Relaunch/Publikationen/coop-infografik-recycling_de.pdf. [Accessed: 19-Feb-2020].

[26] Aldi, "Recycling," 2020. [Online]. Available: https://www.aldi-suisse.ch/de/infos/aldi-suisse-a-z/r/recycling/. [Accessed: 19-Feb-2020].

[27] Swiss Recycling, "Plastikflaschen. Separatsammlung.," 2020. [Online]. Available: http://www.swissrecycling.ch/wertstoffe/plastikflaschen/. [Accessed: 20-Feb-2020].

[28] B. Fischer, "Grundlagenbericht zur Kunststoffsammlung aus Haushalten für den Zweckverband der Zuger Einwohnergemeinden für die Bewirtschaftung von Abfällen (zeba).," 2018. [Online]. Available: https://www.zg.ch/behoerden/weitere-organisationen/zeba/abfallsortierung/wiederverwertbare-abfaelle-1/flyer-pe-sammlung/kunststoffsammlung-aus.pdf. [Accessed: 17-Jun-2020].

[29] T. Pohl, "Übersicht der verschiedenen Sammelsysteme in der Schweiz," Presentation at the BAFU Kunststofftagung, Bern, 2017.

[30] M. Gasser, H. Böni, and P. Wäger, "Gemischte Sammlung von Kunststoffen aus Haushalten. Monitoring der Pilotphase des KUH-Bag Systems.," St. Gallen, 2017.

[31] KUH-Bag, "Kuhl - Easy - Ökologisch. Was gehört in den KUH-Bag.," 2020. [Online]. Available: https://www.kuh-bag.ch/?lid=1#!2. [Accessed: 20-Feb-2020].

[32] Kunststoffsammelsack, “Do`s & Don`t für den Kunststoff-Sammelsack.,” 2020. [Online]. Available: https://www.kunststoffsammelsack.ch/sammeln/sammlung-kunststoff/. [Accessed: 20-Feb-2020].

[33] Sammelsack, “Was gehört in den Sammelsack,” 2020. [Online]. Available: https://www.sammelsack.ch/sammelwertstoffe.html. [Accessed: 20-Feb-2020].

[34] EPS-Verband Schweiz, “EPS Recycling,” 2019. [Online]. Available: http://www.epsschweiz.ch/dokumente/Bro_Recycling_dt.pdf. [Accessed: 30-Oct-2019].

[35] EPS-Verband Schweiz, “Flächendeckendes EPS-Recycling,” 2001. [Online]. Available: http://www.epsschweiz.ch/dokumente/MerkblattEPS.pdf. [Accessed: 21-Feb-2020].

[36] Swiss Recycling, “EPS (Styropor),” 2020. [Online]. Available: http://www.swissrecycling.ch/wertstoffe/eps-styropor/. [Accessed: 21-Feb-2020].

[37] Kuster, “Styropor / Sagex / EPS,” 2020. [Online]. Available: https://www.kuster-recycling.ch/recycling/wertstoffe/styropor-sagex-eps/. [Accessed: 21-Feb-2020].

[38] VSBH, “Diverse Angebote. PE-Recycling.,” 2019. [Online]. Available: https://www.vsbh.ch/de/diverse-angebote/. [Accessed: 09-Jul-2019].

[39] HGC, “Vom Baumateriallager auf die Baustelle - und zurück,” 2019. [Online]. Available: www.hgc.ch/pe-recycling. [Accessed: 17-Jul-2019].

[40] M. Schaub, “Wo landet der separat gesammelte Kunststoff in der Schweiz? Materialflussanalyse von Industrieverpackungen aus Kunststoff in der Schweiz im Jahr 2018». Bachelor thesis. Institute of Environmental Engineering, ETH Zurich.,” 2019.

[41] M. Haupt, C. Vadenbo, and S. Hellweg, “Do We Have the Right Performance Indicators for the Circular Economy?: Insight into the Swiss Waste Management System,” *J. Ind. Ecol.*, vol. 21, no. 3, pp. 615–627, 2017.

[42] Müller Recycling, “Kunststoffrecycling,” 2020. [Online]. Available: https://www.mueller-recycling.ch/fileadmin/user_upload/aktuelles/4_Kunsstoffrecycling.pdf. [Accessed: 29-Jul-2020].

[43] Groupe Barec, “Kunststoffe,” 2020. [Online]. Available: http://www.barec.ch/de/altpapier-kunststoffe/produkte/kunststoffe.html. [Accessed: 29-Jul-2020].

[44] Cand-Landi, “RC-Plast SA,” 2020. [Online]. Available: http://www.candlandi.com/site/fr/activites/recyclage/rc-plast-sa/. [Accessed: 29-Jul-2020].

[45] InnoRecycling, “RESI - Recycling von Silofolien.,” 2020. [Online]. Available: http://www.resi.ch/de/. [Accessed: 21-Feb-2020].

[46] Senn Brunnen, “Entsorgen mit System!,” 2020. [Online]. Available: http://www.senn-brunnen.ch/relog.pdf. [Accessed: 21-Feb-2020].

[47] REDILO, R. Schelker, and P. Geisselhardt, “Erhebung der Kunststoff Mengenströme in der Schweiz (Schwerpunkt Polyolefine): „Stoff-Strom-Atlas Kunststoffe Schweiz“. Bericht 2007,” pp. 1–70, 2008.

[48] R. Geyer, J. R. Jambeck, and K. L. Law, “Production, uses, and fate of all plastics ever made,” *Sci. Adv.*, vol. 3, no. 7, p. 5, 2017.

[49] Schweizerischer Baumeisterverband, “Abfalltrennung auf der Baustelle mit dem Mehr-Mulden-Konzept. Rahmenkonzept.” 2001.

[50] Amt für Umweltschutz des Kantons Schwyz, “Abfalltrennung auf der Baustelle mit dem Mehrmuldenkonzept. Merkblatt.,” 2008.

[51] GEO Partner AG, PVCH, and Abfallfachstellen Ostschweiz/FL, “Merkblatt „Verwertungsmöglichkeiten für PVC-Bauabfälle“,” 2011. [Online]. Available: https://extranet.kvu.ch/files/documentdownload/190916193000_Merkblatt_PVC_Bauabfaelle_Stand_2019_09_16_def.pdf. [Accessed: 02-Mar-2020].

[52] S. Gerassimidou, C. A. Velis, P. T. Williams, M. J. Castaldi, L. Black, and D. Komilis, “Chlorine in waste-derived solid recovered fuel (SRF), co-combusted in cement kilns: A systematic review of sources, reactions, fate and implications,” *Crit. Rev. Environ. Sci. Technol.*, vol. 51, no. 2, pp. 140–186, 2020.

[53] BUWAL, “Entsorgung von Abfällen in Zementwerken. Richtlinie. 2. aktualisierte Auflage. Vollzug Umwelt.,” 2005.

[54] ARP, “Profitieren auch Sie von der sinnvollen Verwertung von PVC-Belägen!,” 2020. [Online]. Available: https://www.arpschweiz.ch/. [Accessed: 02-Mar-2020].

[55] Ecoinvent v. 3.6, “Dataset for ‘window frame production, poly vinyl chloride, U=1.6 W/m2K’ [RER].” 2019.

[56] Stiftung Auto Recycling Schweiz (SARS), “Jahresbericht Stiftung Auto Recycling Schweiz 2017,” 2017.

[57] BAFU, “Altfahrzeuge,” 2020. [Online]. Available: https://www.bafu.admin.ch/bafu/de/home/themen/abfall/abfallwegweiser-a-z/altfahrzeuge.html. [Accessed: 23-Dec-2020].

[58] BAFU, “Erhebung der Kehrichtzusammensetzung, 2012,” pp. 1–63, 2014.

[59] Swico and SENS, “Toocy material flow database.” 2017.

[60] Swico; SENS; SLRS, “Technical Report 2017,” 2017.

[61] TEXAID, “Effiziente Sortierung,” 2020. [Online]. Available: https://www.texaid.ch/de/produkte-leistungen/sortierung.html. [Accessed: 23-Dec-2020].

[62] Tell-Tex, “Containersammlung. Was wir sammeln.,” 2020. [Online]. Available: https://www.tell-tex.ch/de/betrieb/containersammlungen. [Accessed: 09-Apr-2020].

[63] Caritas, “Mit Sachspenden Gutes tun,” 2021. [Online]. Available: https://www.caritas.ch/de/spenden/spenden/waren-spenden.html. [Accessed: 03-Feb-2021].

[64] Caritas, “Kleiderzentrale,” 2021. [Online]. Available: https://www.caritas.ch/de/was-wir-tun/engagement-schweiz/kleiderzentrale.html. [Accessed: 07-Apr-2021].

[65] TEXAID, “Sustainability Report 2017,” 2021. [Online]. Available: https://www.texaid.ch/sustainability-report/. [Accessed: 07-Apr-2021].

[66] Tell-Tex, “Jahresberichte 2018,” 2019. [Online]. Available: https://www.tell-tex.ch/de/ueber-uns/jahresberichte. [Accessed: 12-Dec-2019].

[67] M. Schlummer, A. Maurer, S. Wagner, A. Berrang, T. Fell, and F. Knappich, “Recycling of flame retarded waste polystyrene foams (EPS and XPS) to PS granules free of hexabromocyclododecane (HBCDD),” *Adv. Recycl. Waste Manag.*, vol. 02, no. 02, pp. 6–10, 2017.

[68] J. Woidasky and A. Stolzenberg, “Verwertungspotenzial für Kunststoffteile aus Altfahrzeugen in Deutschland Inhaltsverzeichnis,” 2003.

[69] WIPAG, “Entlackung. Beispiel: Aufbereitung von Altstoßfängern.,” 2020. [Online]. Available: https://www.wipag.de/Kunststoffaufbereitung/Entlackung_id33. [Accessed: 03-Apr-2020].

[70] H. Wilts, N. von Gries, I. Dehne, R. Oetjen-Dehne, N. Buschow, and J. Sanden, “Entwicklung von Instrumenten und Maßnahmen zur Steigerung des Einsatzes von Sekundärrohstoffen – mit Schwerpunkt Sekundärkunststoffe. Report for the Umweltbundesamt. Forschungskennzahl 3712 33 340, UBA-FB 002343.,” 2016.

[71] P. Wäger, R. Hischier, and Müller-Guttenbrunn Group, “Life cycle assessment of post-consumer plastics production from waste electrical and electronic equipment (WEEE) treatment residues in a Central European plastics recycling plant.,” 2020. [Online]. Available: https://www.mgg-recycling.com/wp-content/uploads/LCA-MBA-Polymers-Austria.pdf. [Accessed: 28-Sep-2020].

[72] G. Sandin and G. M. Peters, “Environmental impact of textile reuse and recycling – A review,” *J. Clean. Prod.*, vol. 184, pp. 353–365, 2018.

[73] Å. Östlund *et al.*, *Textilåtervinning. Tekniska möjligheter och utmaningar. Rapport 6685.* 2015.

[74] PVCH, “Pressemitteilung. Rücknahme und Recycling von PVC-Rohren gestartet.,” 2008. [Online]. Available: https://www.abfall.ch/pages/info/pdf/Pressemitteilung_final.pdf. [Accessed: 02-Mar-2020].

[75] Kunststoffrohrverband e.V. (KRV), “Recycling,” 2020. [Online]. Available: https://www.krv.de/wissen/recycling. [Accessed: 02-Mar-2020].

[76] Tönsmeier, “Annahmekatalog. Kompetenzbereich Kunststoffrohre.,” 2020. [Online]. Available: https://www.toensmeier.com/fileadmin/toensmeier/Bedingungen/Annahmekatalog_Kunststoffrohre.pdf. [Accessed: 29-Jul-2020].

[77] Kunststoffrohrverband e.V. (KRV), “Recycling von PVC-U,” 2020. [Online]. Available: https://www.krv.de/wissen/recycling-von-pvc-u. [Accessed: 17-Oct-2020].

[78] Ziegelgut Recycling, “Folien,” 2020. [Online]. Available: http://www.zirec.ch/folien.htm. [Accessed: 02-Mar-2020].

[79] F. Leimgruber, “Autorecycling : Stand der Technik und Herausforderungen,” Presentation at the Recyclingkongress, Biel, 2019.

[80] SPI, “Automotive Recycling. Devalued is now Revalued,” 2016.

[81] MBA Polymers, “Our Locations. Production Unit United Kingdom.,” 2020. [Online]. Available: https://mbapolymers.com/company/locations/#united-kingdom. [Accessed: 05-Aug-2020].

[82] A. Buekens and X. Zhou, “Recycling plastics from automotive shredder residues: A review,” *J. Mater. Cycles Waste Manag.*, vol. 16, no. 3, pp. 398–414, 2014.

[83] Paul Scherrer Institut, “RESH Behandlung mit KVAplus. Management Summary,“ 2009.

[84] D. Lucas *et al.*, “Methods of Responsibly Managing End-of-Life Foams and Plastics Containing Flame Retardants: Part I,” *Environ. Eng. Sci.*, vol. 35, no. 6, pp. 573–587, 2018.

[85] emr, “Our journey towards ZERO WASTE.,” 2020. [Online]. Available: http://uk.emrgroup.com/zero-waste.php. [Accessed: 05-Aug-2020].

[86] Galloo Plastics, “The Company.,” 2020. [Online]. Available: https://gallooplastics.eu/en/the-company/. [Accessed: 05-Aug-2020].

[87] D. Lucas *et al.*, “Methods of Responsibly Managing End-of-Life Foams and Plastics Containing Flame Retardants: Part II,” *Environ. Eng. Sci.*, vol. 35, no. 6, pp. 588–602, 2018.

[88] AWEL, “Fahrzeug- und Reifenverwertung.,” 2020. [Online]. Available: https://awel.zh.ch/internet/baudirektion/awel/de/betriebe_anlagen_baustellen/abfallanlagen/fahrzeug_reifenverwertung.html. [Accessed: 05-Aug-2020].

## 4. Stakeholders personal communication

| Surname | First name | Affiliation |
|---|---|---|
| Aebersold | Adrian | Zürcher Abfallverwertungs AG (ZAV), head of market management |
| Arz | Karin | ROOFCOLLECT, general manager |
| Bachmann | Dörte | SV Group, sustainability manager |
| Bachmann | Mirko | Forbo-Giubiasco SA, head of marketing and application technology |
| Bieler | Patric | Syngenta, head of global process technology new active ingredients |
| Burkhalter | Markus | Canplast, sales |
| Dalla Bona | Marco | EPS-Verband Schweiz, president |
| Dewulf | Jo | Ghent University, full professor |
| Diebold | Frank | swisswindows, building physicist, development and technology |
| Fuchs | Guido | Coop, project manager sustainability |
| Gasser | Michael | EMPA, research associate, recycling |
| Geisselhardt | Patrik | Swiss Recycling, managing director |
| Helminiak | Norbert | ARP Schweiz, managing director |
| Hiltbrunner | Martin | Hiltbrunner AG, business owner |
| Huhn | Armin | Schneider Umweltservice, head of recycling |
| Hummen | Torsten | Bosch, design for environment specialist |
| Kalberer | Andreas | Agroscope, researcher |
| Kaufmann | Andreas | VASSO, president |
| Kawecki | Delphine | EMPA, researcher |
| Küchler | Daniel | HG Commerciale, head of marketing |
| Leimgruber | Frédéric | Thévenaz-Leduc SA, sales manager |
| Magnenat | Raphaël | Recovinyl, regional representative for Switzerland, Hungary, Romania and Bulgaria |
| Meier | Trix | EPS Verband Schweiz, managing director |
| Müller | Thomas | Müller Recycling AG, operations manager |
| Nadler | André | emüller, managing director |
| Oswald | Heidi | Migros-Genosseschafts-Bund, project leader environment |
| Röschli | Kurt | Swiss Plastics, managing director |
| Scharff | Christoph | Altstoff Recycling Austria AG (ARA), board spokesman |
| Schaub | Andreas | SIKA Supply Center Sarnen, authorized representative |
| Senn | Martin | Senn Brunnen AG, company owner |
| Stalder | Josef | Bieberbau, head of department windows |
| Staubli | Roger | Sortag |
| Steinauer | Edgar | Steinauer AG Recycling und Umweltservice, chairman of the board |
| Stotz | Julian | Coop, packaging engineer |
| Tonner | Markus | InnoRecycling AG, managing director |
| Turcot | Zoé | Migros-Genosseschafts-Bund, junior project leader sustainability |
| Van der Haegen | Patric | Eberhard Unternehmungen, division head development |

| | | |
|---|---|---|
| Vetter | Michael | Rewindo, managing director |
| Wiederkehr | Peter | Wiederkehr Recycling AG, company owner |
| Würmli | Jean-Claude | Verein PET-Recycling Schweiz, managing director |
| Wyder | Lukas | Verband des Schweizerischen Baumaterial-Handels (VSBH), managing director |
| Zimmermann | Jochen | Arbeitsgemeinschaft PVC-Bodenbelag Recycling (AgPR), managing director |
