# Supplementary figures and images for: A high-resolution dataset on the plastic material flows in Switzerland
Source: Data Brief. 2022 Mar 2;41:108001. doi: 10.1016/j.dib.2022.108001 (PMC8914542; doi:10.1016/j.dib.2022.108001)

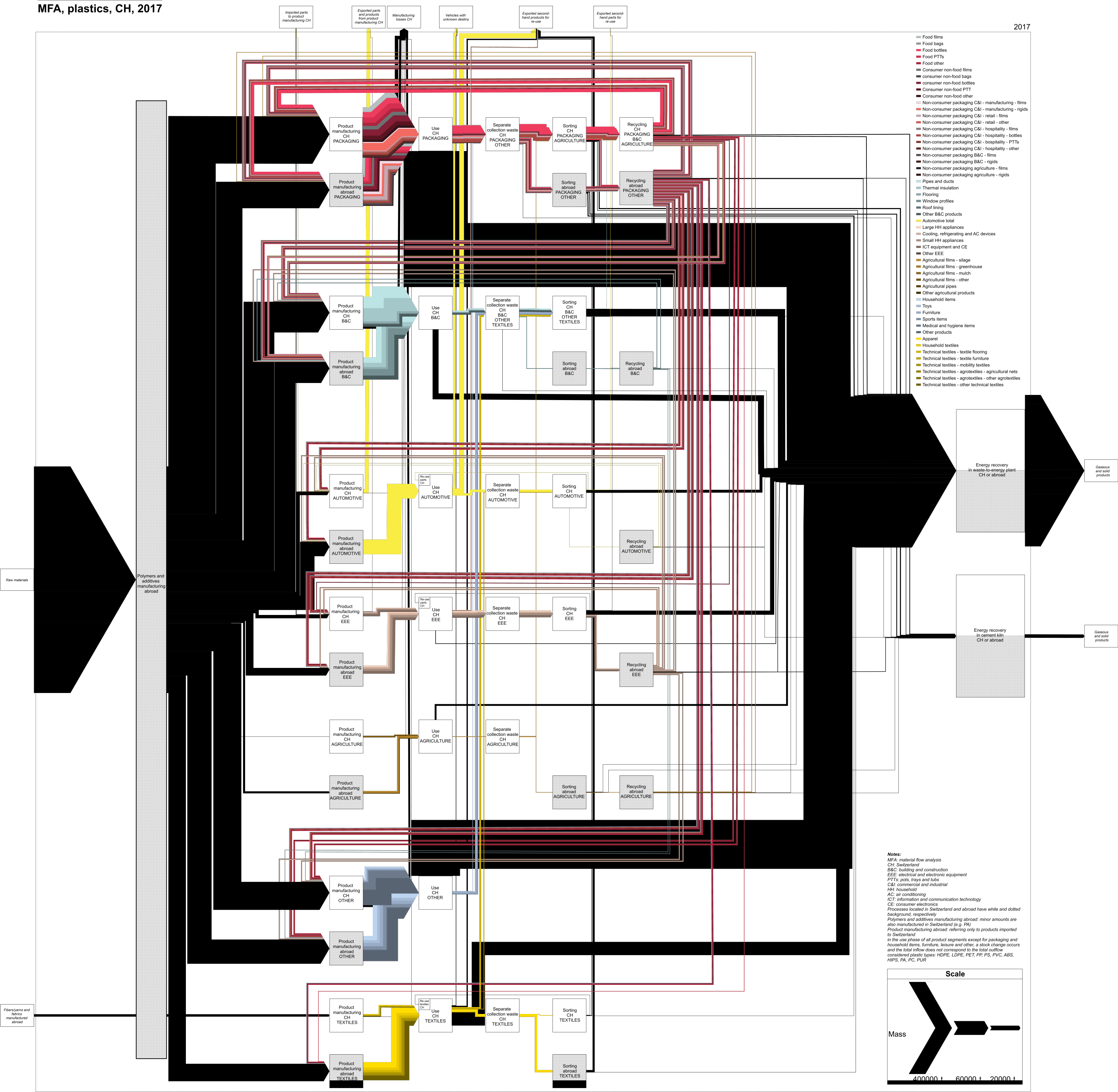

Supplement: Supplementary file 5 [file mmc5.pdf]
